# Genomic Epidemiology Insights on NDM-Producing Pathogens Revealed the Pivotal Role of Plasmids on *bla*_NDM_ Transmission

**DOI:** 10.1128/spectrum.02156-21

**Published:** 2022-02-28

**Authors:** Huiyue Dong, Yan Li, Jing Cheng, Ziwei Xia, Wentian Liu, Tingting Yan, Fangfang Chen, Zhiqiang Wang, Ruichao Li, Jinjin Shi, Shangshang Qin

**Affiliations:** a School of Pharmaceutical Sciences, Key Laboratory of Advanced Drug Preparation Technologies, Ministry of Education, Zhengzhou University, Zhengzhou, Henan, China; b Jiangsu Co-Innovation Center for Prevention and Control of Important Animal Infectious Diseases and Zoonoses, College of Veterinary Medicine, Yangzhou University, Yangzhou, Jiangsu, China; c Institute of Comparative Medicine, Yangzhou University, Yangzhou, Jiangsu, China; Labcorp

**Keywords:** *Enterobacterales*, plasmid diversity, *bla*_NDM_, molecular epidemiology, nanopore sequencing

## Abstract

Incidences of nosocomial infections mediated by New Delhi metallo-β-lactamase (NDM) enzyme-producing *Enterobacterales* are increasing globally, resulting in a great burden to public health. The carbapenem-resistant *Enterobacterales* (CRE) were collected from Henan, China during 2013–2016. The *bla*_NDM_-positive strains were characterized using PCR, antimicrobial susceptibility testing, conjugation assay, S1 nuclease pulsed-field gel electrophoresis (S1-PFGE), Southern blot, whole-genome sequencing (WGS), and bioinformatics analysis. Eighty-one NDM-producing strains were identified among 391 nonduplicate CRE strains. Among them, four strains cocarried *mcr* and *bla*_NDM_ genes, and two carried *bla*_IMP-4_ and *bla*_NDM_ genes. The coexistence of *bla*_NDM-5_ and *mcr-9* in Enterobacter hormaechei was found for the first time. In total, four *bla*_NDM_ subtypes were identified. Among them, *bla*_NDM-1_ and *bla*_NDM-5_ were predominant. There was an obvious increasing trend in *bla*_NDM-5_ from 2013 to 2016. Thirteen different bacterial species were found among the 81 strains, and Escherichia coli was the dominant strain. *bla*_NDM_ genes were located on nine different Inc-type plasmids, most of them on the IncX3 plasmids, except for the Pr-15-2-50 strain, which was located on the chromosome. We characterized two novel plasmids: the IncHI5-like plasmid carrying *bla*_NDM-9_ found in *K. pneumonia*, and the IncI1 *bla*_NDM-5_-positive plasmid. These findings provide the genomic basis for the widespread transmission of *bla*_NDM_ and pave the way for the formulation of more effective monitoring and control methods.

**IMPORTANCE** To control the emergence and transmission of CRE, it is important to perform retrospective genomic investigations. It is important to evaluate the plasmid diversity, genetic environment, and evolutionary relationships of the *bla*_NDM_-positive clinical strains in the early transmission stages. This study conducted an in-depth analysis of *bla*_NDM_-positive pathogens during a 4-year period using different methods for observing the high prevalence and active transmission of *bla*_NDM_-positive CRE. Moreover, we also explored the coexistence of the *bla*_NDM_ and *mcr,* a clinically important mobile colistin resistance gene. This study shows that the prevalence of *bla*_NDM_-positive pathogens in Henan is high and the isolation rates increase each year. Moreover, plasmid-mediated horizontal transfer plays an important role in *bla*_NDM_ dissemination. The co-occurrence of multiple resistance genes highlighted a long-lasting evolutionary pathway. Therefore, we have suggested the long-term continuous surveillance of clinical pathogens carrying *bla*_NDM_ to learn the future transmission trend and curb the public health risk caused by CRE.

## INTRODUCTION

Carbapenem antibiotics are β-lactam antibiotics with a broad antibacterial spectrum and strong antibacterial activity. They are the most important antibiotics for the treatment of multidrug-resistant (MDR) Gram-negative bacterial infections (1). However, the clinical use of these drugs leads to the emergence of carbapenem-resistant *Enterobacterales* (CRE) (2) and makes clinical medication selection difficult. In 2013, the Centers for Diseases Control and Prevention in the U.S. reported that more than 9,000 health care-related infections were caused by CRE each year. It ranked CRE in the highest threat level. Moreover, the China CRE Monitoring Network showed that the hospital mortality rate of CRE was 33.5% (222/662) (3). It also showed that the mortality rate increased with the length of hospital stay.

Carbapenem-inactivating carbapenemases are predominantly divided into Classes A, B, and D according to the Ambler classification. Classes A and D belong to serine enzymes, and B belongs to metallo-β-lactamases (MBLs). NDM is a typical member of the B1 class of MBLs. It is capable of hydrolyzing all β-lactams, except monobactams (4). It recruits mobile genetic elements, such as plasmids belonging to different replicon or Inc types (IncFII, IncHI2, IncN, and IncX3), insertion sequences (IS*Aba125*, IS*CR1*), and transposons (Tn*125*) (5). *bla*_NDM_ genes have already spread to various species of bacteria worldwide, including *Enterobacterales* and nonfermenting Gram-negative bacilli (6). The increasing prevalence of NDM-producing pathogens has seriously compromised the efficacy of carbapenems in clinical settings, and it poses a great threat to public health. According to current reports, 28 NDM variants have been identified in multiple species of *Enterobacterales*, Acinetobacter, and Pseudomonas. NDM-1 and NDM-5, which were encoded mainly by IncX3 plasmids, were the most frequently detected variants in *Enterobacterales*. However, NDM-5 was more prevalent compared to NDM-1 in Escherichia coli. Our previous study revealed only NDM-1, and no other variants were detected in NDM-producing *Enterobacterales* isolated from the Henan province between 2011 and 2012. Moreover, the IncA/C plasmids with broad-host-range were the predominant vehicles for *bla*_NDM_ compared to the narrow-host range IncX3 plasmids (7). These differences indicate the changes in the prevalence and evolution of *bla*_NDM_-bearing plasmids. Therefore, we continuously monitored the NDM-producing CRE strains in a teaching hospital in Zhengzhou University over a 4-year period (2013–2016). We tried to elucidate the molecular mechanisms for the *bla*_NDM_ gene transfers, and study the evolution of the epidemic *bla*_NDM_ plasmids and their clones.

## RESULTS

### Overview of NDMs-producing CRE isolates.

From 2013 to 2016, 391 nonduplicate CRE isolates belonging to 13 different species were collected from a teaching hospital in the Zhengzhou University for screening carbapenemase genes using PCR and Sanger sequencing. The result showed 291 Klebsiella pneumoniae strains (74.42%) carrying the *bla*_KPC-2_ gene and another 81 (20.72%) belonging to various species carrying the *bla*_NDM_ (Table 1). This illustrated that K. pneumoniae and E. coli were the main clinical CREs. KPC and NDM were the primary carbapenem-inactivating enzymes in CRE recovered from the Henan province. It was well recognized that *bla*_NDM_ genes were mainly carried by Gram-negative *Enterobacterales*, including E. coli, K. pneumoniae, Citrobacter freundii, and Enterobacter cloacae (8–10). The prevalence of *bla*_NDM_ in different *Enterobacterales* was 49.38% (40/81), 14.81% (12/81), 13.58% (11/81), 7.41% (6/81), and 4.94% (4/81) in E. coli, K. pneumoniae, Enterobacter hormaechei, C. freundii, and Citrobacter portucalensis, respectively. There was also 1.23% (1/81) in each Citrobacter braakii, Klebsiella aerogenes, Klebsiella pasteurii, Klebsiella oxytoca, Raoultella ornithinolytica, Serratia marcescens, Proteus mirabilis, and Providencia rettgeri. This indicates that E. coli was the most common host for *bla*_NDM,_ followed by K. pneumoniae and E. hormaechei. Sanger sequencing of *bla*_NDM_ genes identified four *bla*_NDM_ subtypes, including *bla*_NDM-1_ (*n* = 41), *bla*_NDM-5_ (*n* = 38), *bla*_NDM-4_ (*n* = 2), and *bla*_NDM-9_ (*n* = 1) (Table 1). Among them, *bla*_NDM-5_ was the most prevalent subtype in E. coli (33/40, 82.5%), and the majority of K. pneumoniae carried *bla*_NDM-1_ (9/12, 75%). However, carbapenemase gene *bla*_IMP-4_ was only detected in two NDM-producing strains (KA-14-61 and KO-14-71).

**TABLE 1:** Basic information of the 81 *bla*_NDM_-bearing strains

Isolate	Species	MLST^*a*^	Collection date	Age/sex	Specimen type	Ward	Prognosis	Conjugation frequency	NDM-type	Plasmid type carrying *bla*_NDM_	NDM-positive plasmid size (kb)	Grouping of IncX3 *bla*_NDM_-positive plasmids
KP-13-8	K. pneumoniae	ST494	2013.01.06	61yr/female	blood	Gastroenterology dept	discharge	-^*b*^	NDM-5	IncX3	46	B
EC-13-1	E. coli	ST40	2013.01.25	6days/male	blood	ICU	discharge	-	NDM-1	IncX3	54	B
KP-13-11	K. pneumoniae	ST35	2013.04.25	2mo/female	sputum	ICU	death	3.6 × 10^−4^	NDM-1	IncX3	54	C
CR-13-12	*E. hormaechei*	ST419	2013.05.06	89yr/female	sputum	ICU	discharge	3.3 × 10^−4^	NDM-1	IncFII	87	-
EC-13-22	E. coli	ST361	2013.08.05	41yr/female	drainage liquid	gynecology	discharge	2.6 × 10^−6^	NDM-1	IncC	213	-
EC-13-31	E. coli	ST167	2013.09.04	68yr/male	blood	gynecology	discharge	-	NDM-5	IncX3	46	B
ECL-13-2	*E. hormaechei*	ST177	2013.09.04	53yr/female	urine	urology	discharge	1.8 × 10^−7^	NDM-1	IncFII-IncFIB	138	-
EC-13-30	E. coli	ST167	2013.09.18	35yr/male	secreta	endocrinology	discharge	-	NDM-5	IncX3	46	B
KP-13-7	K. pneumoniae	ST1	2013.09.26	37yr/male	bile	hepatological surgery	discharge	1.33 × 10^−4^	NDM-1	IncX3	54	C
EC-13-33	E. coli	ST540	2013.10.06	68yr/male	blood	gynecology	discharge	3 × 10^−4^	NDM-1	IncFII-IncN	78	-
ECL-13-4	*E. hormaechei*	ST88	2013.10.17	48yr/male	blood	ICU	death	2.8*10^−4^	NDM-5	IncX3	46	B
CF-13-34	*C. portucalensis*	ST328	2013.10.19	23yr/male	secreta	hematology	death	1.1 × 10^−4^	NDM-1	IncX3	54	E
EC-13-49	E. coli	ST167	2013.11.07	78yr/female	urine	kidney internal	discharge	3.5 × 10^−6^	NDM-1	IncC	215	-
ECL-13-37	*E. hormaechei*	ST231	2013.11.14	37yr/male	urine	urology	discharge	-	NDM-5	IncX3	46	B
KP-13-14	K. pneumoniae	ST782	2013.11.23	21days/male	wound	pediatric surgery	discharge	-	NDM-9	IncHI5	358	-
CR-13-36	*E. hormaechei*	ST419	2013.12.05	47yr/female	urine	kidney internal	discharge	1.2 × 10^−5^	NDM-1	IncFII	87	-
PM58	P. mirabilis	NA	2013.12.15	3yr/female	urine	rehabilitation medicine	discharge	9.4 × 10^−6^	NDM-1	-	85	-
KP-14-2-131	K. pneumoniae	ST345	2014.01.23	44yr/male	urine tube tip	neurosurgery	discharge	1.4 × 10^−6^	NDM-1	IncHI5	358	-
KOR-14-72	*R. ornithinolytica*	NA	2014.02.15	71yr/female	sputum	ICU	discharge	3.9 × 10^−5^	NDM-1	IncX3	46	C
KO-14-71	*K. pasteurii*	NA	2014.02.20	67yr/female	sputum	ICU	discharge	3.2 × 10^−3^	NDM-1	IncX3	54	C
EC-14-2-77	E. coli	ST410	2014.03.30	66yr/male	drainage liquid	hepatological surgery	discharge	2.5 × 10^−3^	NDM-4	IncX3	54	C
ECL-14-58	*E. hormaechei*	ST177	2014.05.12	10yr/male	pus	pediatric surgery	discharge	9.5 × 10^−5^	NDM-1	IncX3	54	C
ECL-14-60	*E. hormaechei*	ST696	2014.06.05	62yr/male	blood	ICU	death	-	NDM-1	IncC, IncX3	171-54	D
EC-14-55	E. coli	ST410	2014.06.06	14yr/female	blood	Pediatric medicine	death	-	NDM-4	IncX3	46	C
KA-14-61	K. aerogenes	NA	2014.08.30	33yr/male	secreta	Department of Burn Repair and Reconstruction	discharge	2.2 × 10^−4^	NDM-1	IncX3	46	B
EC-14-54	E. coli	ST167	2014.08.30	51yr/male	sanies	intestine surgery	discharge	-	NDM-5	IncX3	46	B
CF-14-50	C. freundii	ST22	2014.09.20	44yr/male	urine	urology	discharge	1.9 × 10^−4^	NDM-1	IncX3	54	D
ECL-14-56	*E. hormaechei*	ST171	2014.11.02	45yr/male	blood	ICU	death	-	NDM-1	IncX3	54	C
KP-14-6	K. pneumoniae	ST76	2014.11.13	10days/female	blood	Infectious disease	discharge	2.9 × 10^−4^	NDM-1	IncC	200	-
EC-14-2-134	E. coli	ST101	2014.11.17	31yr/male	swab	Burn Repair and Reconstruction	discharge	5.9 × 10^−5^	NDM-5	IncX3	46	B
EC-14-2-92	E. coli	ST167	2014.11.27	50yr/male	blood	oncology	death	4 × 10^−5^	NDM-5	IncX3	46	B
EC-14-2-94	E. coli	ST167	2014.12.10	44yr/female	urine	urology	discharge	-	NDM-5	IncX3	46	B
EC-14-2-9	E. coli	ST167	2014.12.19	51yr/female	sputum	Rheumatology	discharge	-	NDM-5	IncX3	46	B
EC-15-2-5	E. coli	ST167	2015.01.16	26yr/male	sputum	ICU	death	-	NDM-5	IncX3	54	D
EC-15-2-14	E. coli	ST2083	2015.01.25	23yr/female	sputum	Rheumatology	discharge	-	NDM-5	IncX3	46	B
CF-15-43	*C. portucalensis*	ST17	2015.02.21	47yr/female	urine	urology	discharge	3 × 10^−5^	NDM-1	IncX3	54	E
CF-15-2-98	*C. portucalensis*	ST17	2015.02.21	47yr/female	urine	urology	discharge	6.8 × 10^−5^	NDM-1	IncX3	54	E
KP-15-2-113	K. pneumoniae	ST1083	2015.03.08	2mo/male	sputum	neonatology	discharge	3.3 × 10^−5^	NDM-1	IncX3	46	C
EC-15-10	E. coli	ST540	2015.03.14	74yr/male	sputum	ICU	discharge	-	NDM-5	IncX3	54	D
EC-15-3	E. coli	ST6388	2015.03.23	53yr/female	urine	urology	discharge	-	NDM-1	IncFII	110	-
CF-15-61	C. freundii	ST22	2015.04.08	75yr/female	drainage liquid	gastrointestinal surgery	discharge	1.7 × 10^−4^	NDM-1	IncX3	54	C
CF-15-33	C. freundii	NA	2015.05.20	7yr/male	blood	Pediatric medicine	discharge	1.7 × 10^−5^	NDM-1	IncX3	54	C
EC-15-34	E. coli	ST746	2015.05.22	60yr/male	blood	urology	death	-	NDM-5	IncX3	46	B
KP-15-35	K. pneumoniae	ST17	2015.05.22	10days/male	blood	neonatology	death	1.4 × 10^−5^	NDM-1	IncX3	54	C
EC-15-2-35	E. coli	ST540	2015.06.27	75yr/female	urine	urology	discharge	-	NDM-5	IncX3	54	D
EC-15-2-56	E. coli	ST167	2015.06.27	52yr/male	urine	urology	discharge	-	NDM-5	IncX3	46	A
EC-15-2-24	E. coli	ST540	2015.06.29	75yr/female	urine	urology	discharge	2.5 × 10^−5^	NDM-5	IncX3	54	D
EC-15-2-47	E. coli	ST540	2015.06.29	67yr/male	urine	urology	discharge	-	NDM-5	IncX3	54	D
SM-15-2-16	S. marcescens	NA	2015.07.01	27yr/female	sputum	respiratory medicine	discharge	2.5 × 10^−5^	NDM-1	IncX3	46	B
EC-15-2-132	E. coli	ST410	2015.07.15	38ye/female	blood	Hematology dept	discharge	-	NDM-5	IncX3	46	D
EC-15-2-51	E. coli	ST617	2015.07.21	9mo/male	urine	ICU	discharge	-	NDM-5	IncX3	46	B
EC-15-2-65	E. coli	ST6388	2015.07.30	65yr/male	urine	ICU	discharge	6.8 × 10^−5^	NDM-5	IncX3	46	C
KP-15-2-62	K. pneumoniae	ST490	2015.07.30	2yr/female	blood	ICU	death	-	NDM-5	IncX3	46	D
KP-15-2-52	K. pneumoniae	ST1440	2015.08.03	66yr/male	urine	urology	death	5.5 × 10^−6^	NDM-5	IncX3	46	B
CF-15-2-29	C. freundii	ST22	2015.08.04	1mo/female	sputum	ICU	discharge	8.8 × 10^−4^	NDM-1	IncX3	54	C
EC-15-2-26	E. coli	ST167	2015.08.08	49yr/female	urine	gynecology	discharge	-	NDM-5	IncX3	46	B
Pr-15-2-50	P. rettgeri	NA	2015.08.13	19yr/female	joint fluid	internal medicine	discharge	-	NDM-1	-	-	-
EC-15-2-1	E. coli	ST167	2015.10.28	67yr/female	urine	cardiac surgery	discharge	-	NDM-5	IncX3	54	B
EC-15-2-2	E. coli	ST617	2015.10.28	56yr/male	drainage liquid	hepatological surgery	discharge	3.3 × 10^−5^	NDM-5	IncX3	54	D
KP-15-2-6	K. pneumoniae	ST11	2015.11.05	78yr/male	sputum	respiratory medicine	discharge	1.1 × 10^−5^	NDM-1	IncX3	54	C
EC-15-2-152	E. coli	ST405	2015.12.02	59yr/female	blood	ICU	death	-	NDM-5	IncX3	46	B
EC-15-2-153	E. coli	ST405	2015.12.04	61yr/male	drainage liquid	gastrointestinal surgery	discharge	-	NDM-1	IncX3	46	B
EC-15-2-159	E. coli	ST167	2015.12.08	23yr/male	urine	urology	discharge	-	NDM-5	IncX3	46	B
CF-15-2-165	C. portucalensis	NA	2015.12.11	79yr/male	urine	urinary surgery	discharge	3.3 × 10^−4^	NDM-1	IncX3	54	E
EC-16-7	E. coli	ST167	2016.01.08	52yr/female	urine	kidney internal	discharge	4.3 × 10^−4^	NDM-1	IncX3	54	B
ECL-16-5	E. hormaechei	ST51	2016.01.08	82yr/male	sputum	ICU	death	3.3 × 10^−4^	NDM-1	IncX3	54	B
EC-16-10	E. coli	ST1193	2016.03.03	79yr/male	blood	ICU	death	-	NDM-5	IncI1	93	-
CF-16-17	C. freundii	ST18	2016.07.08	70yr/male	secreta	endocrinology	discharge	1.8 × 10^−5^	NDM-1	IncX3	54	E
KO-16-21	K. oxytoca	NA	2016.07.10	83yr/male	sputum	ICU	discharge	3.8 × 10^−5^	NDM-1	IncHI5	370	-
EC-16-20	E. coli	ST617	2016.07.10	48yr/male	ascites	Infectious disease	discharge	-	NDM-5	IncX3	46	B
EC-16-35	E. coli	ST167	2016.07.16	10yr/female	ascites	pediatric surgery	discharge	3.2 × 10^−5^	NDM-5	IncX3	46	B
EC-16-37	E. coli	ST46	2016.07.18	51yr/female	urine	urology	discharge	1.5 × 10^−6^	NDM-5	IncFII-IncFIA-IncFIB	159	-
EC-16-52	E. coli	ST410	2016.07.21	63yr/female	urine	pediatric surgery	discharge	-	NDM-5	IncX3	46	B
KP-16-57	K. pneumoniae	ST716	2016.07.26	10yr/male	sputum	ICU	discharge	7.3 × 10^−4^	NDM-1	IncC	180	-
CF-16-58	C. braakii	NA	2016.07.27	57yr/male	urine	respiratory medicine	discharge	5.9 × 10^−4^	NDM-1	IncX3	54	C
EC-16-59	E. coli	ST167	2016.07.29	45yr/male	tissue	kidney internal	discharge	-	NDM-5	IncX3	46	B
EC-16-60	E. coli	ST167	2016.07.29	2mo/female	sputum	ICU	discharge	6.3 × 10^−4^	NDM-5	IncX3	54	C
CF-16-61	C. freundii	ST22	2016.07.30	50yr/male	blood	ICU	discharge	8.8 × 10^−5^	NDM-1	IncX3	54	C
ECL-16-74	E. hormaechei	ST93	2016.08.03	45yr/male	drainage liquid	Liver transplantation	discharge	-	NDM-5	IncX3	46	C
EC-16-76	E. coli	ST2172	2016.08.06	58yr/male	urine	emergency internal medicine	discharge	-	NDM-5	IncX3	54	C
ECL-16-79	E. hormaechei	ST51	2016.10.20	54yr/female	bile	intervention department	discharge	8.1 × 10^−4^	NDM-5	IncX3	46	B

a MLST, multilocus sequence typing; NA, not available.

b -, not detected.

**FIG 1:**
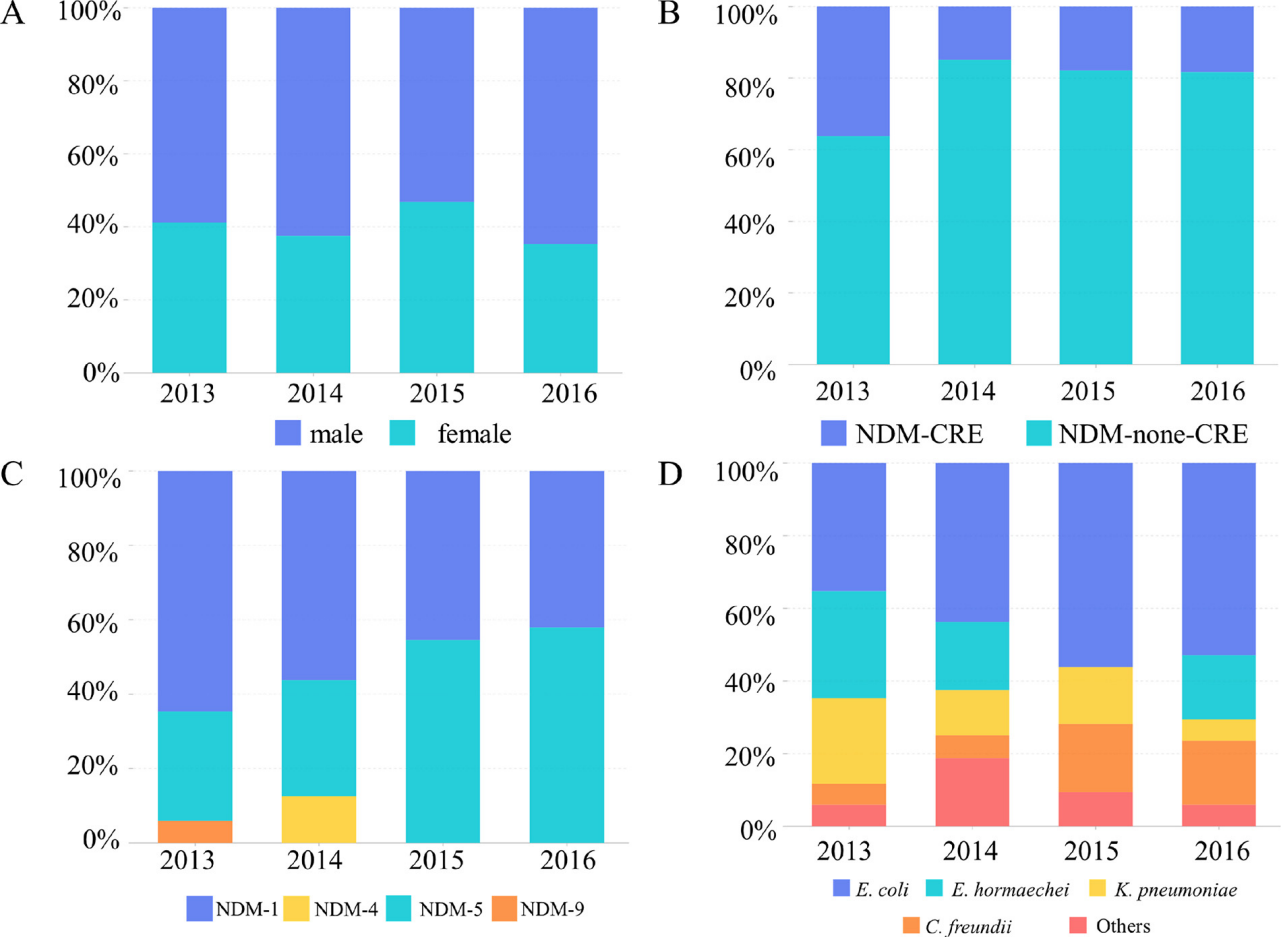
Epidemiological description and impact factors of the 81 *bla*_NDM_-positive strains used in this study. (A) The proportion carrying the NDM by gender in different years. (B) Isolation rates of NDM among CRE in different years. (C) Proportion of NDM subtypes isolated in different years. (D) Proportion of different species isolated in different years.

We analyzed the clinical features of these 81 *bla*_NDM_ carriers (Table 1). We found that most *bla*_NDM_-positive strains were isolated from medical Intensive Care Units (ICUs). ICU patients usually have longer hospital stays, which increases the risk of infections and evolution of CRE pathogens. Comparatively, higher NDM-positive rates were also obtained among the Urinary Surgery and Pediatrics wards. The number of male patients was slightly higher compared with female patients (Fig. 1). We observed a wide age gap among these patients, ranging from 6 days to 89 years old; however, maximum cases (49.38%) were concentrated in the 50–79 age group. The mortality among the NDM-positive patients was 18.52%, which was lower compared to our previous report (7).

### Resistance phenotype, determinants, and bacterial genotyping.

**FIG 2:**
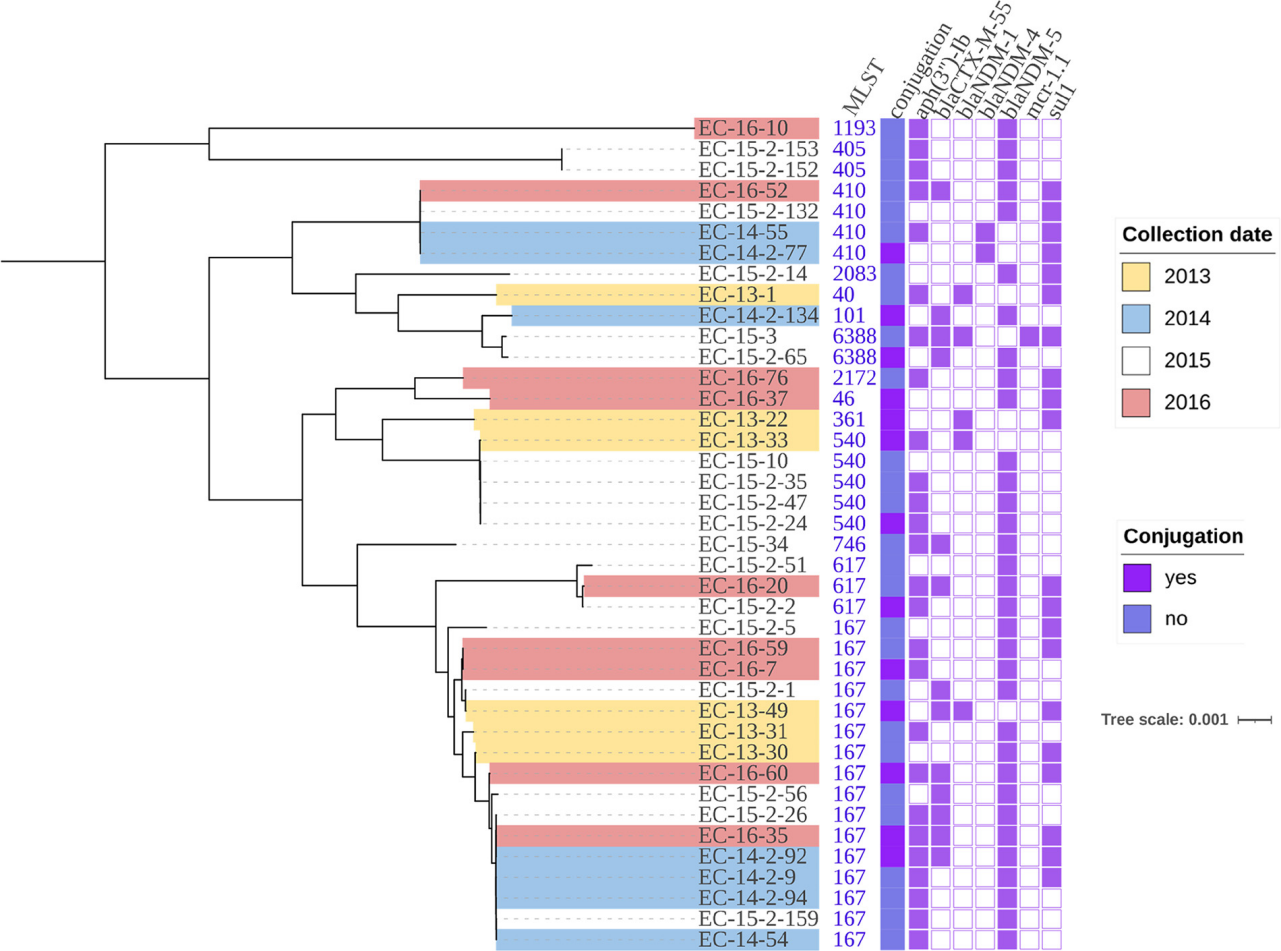
Phylogenetic tree of all 40 *bla*_NDM_-positive E. coli isolates from 2013–2016. Resistance genes are indicated by squares: solid square indicates has; hollow square indicates does not have.

Antimicrobial susceptibility testing revealed that all the 81 *bla*_NDM_-positive strains were MDR strains, and they were resistant to multiple categories of antimicrobials (*n* ≥ 3) (Table S2 in the supplemental material). Therefore, each isolate carried at least three categories of resistance genes associated with the resistance phenotype (Fig. 2 and Fig. S1). The MIC values of meropenem or imipenem were distributed between 16 and 64 μg/mL. Given that most NDM-producing isolates (92.59%) were resistant to aztreonam, we detected β-Lactamase encoding genes other than carbapenemase. Therefore, various AmpC (CMY, ACT, DHA) and ESBL (CTX-M, TEM, SHV, VEB, SFO, OXA) genes were identified in different species (Fig. 2 and Fig. S1). Moreover, four strains (EC-15-3, CF-15-2-29, ECL-16-5, and ECL-16-79) also contained plasmid-borne colistin resistance genes (*mcr-1* or *mcr-9*). The abundance of antibiotic resistance genes in strains increases the risk of *bla*_NDM_ cotransmission. To evaluate the transferability of *bla*_NDM_ genes, conjugation assays were performed for the 81 *bla*_NDM_-positive strains with E. coli (EC600 or J53). The *bla*_NDM_ genes carried by 46 strains were successfully transferred to the recipient, suggesting that the *bla*_NDM_ genes carried by these 46 strains were located in conjugative plasmids or other mobilizable genetic elements. The conjugation frequencies ranged from 2.5 × 10^−3^ to 1.8 × 10^−7^ (Table 1).

As the most abundant species carrying *bla*_NDM_, evolutionary relationships between the 40 E. coli isolates were investigated and a phylogenetic tree based on SNPs of the core genome data was constructed (Fig. 2). These isolates were assigned to 14 distinct sequence types (STs), and ST167 (16/40, 40%) was the most prevalent ST (Table 1). This finding was in agreement with the previous results (11), which suggest that ST167 appears to be the predominant type of *bla*_NDM_-positive E. coli in China. To further investigate the evolutionary relationship between these ST167 E. coli and other ST167 E. coli collected from the NCBI database (Table S3), a phylogenetic tree based on SNPs of the core genomes was constructed. ST167 E. coli carrying *bla*_NDM_ were mainly found in humans. However, they are also found in pets and environmental samples (Fig. S2). *bla*_NDM-5_ was dominant in this subtype. Observation of diverse STs in E. coli indicated plasmids or other horizontal mobile elements to be considered as the main vehicles for *bla*_NDM_ transmission. Similarly, four STs were identified among the eight C. freundii. Moreover, K. pneumoniae (*n* = 12) and *E. hormaechei* (*n* = 11) contained 12 and 8 different STs, respectively. The wide distribution of NDM-producing strains illustrates that in inter- and intraspecies, horizontal gene transfer plays the most important role in the transmission of *bla*_NDM_ genes.

### Systematic analysis of the predominant IncX3 *bla*_NDM_-bearing plasmids.

**FIG 3:**
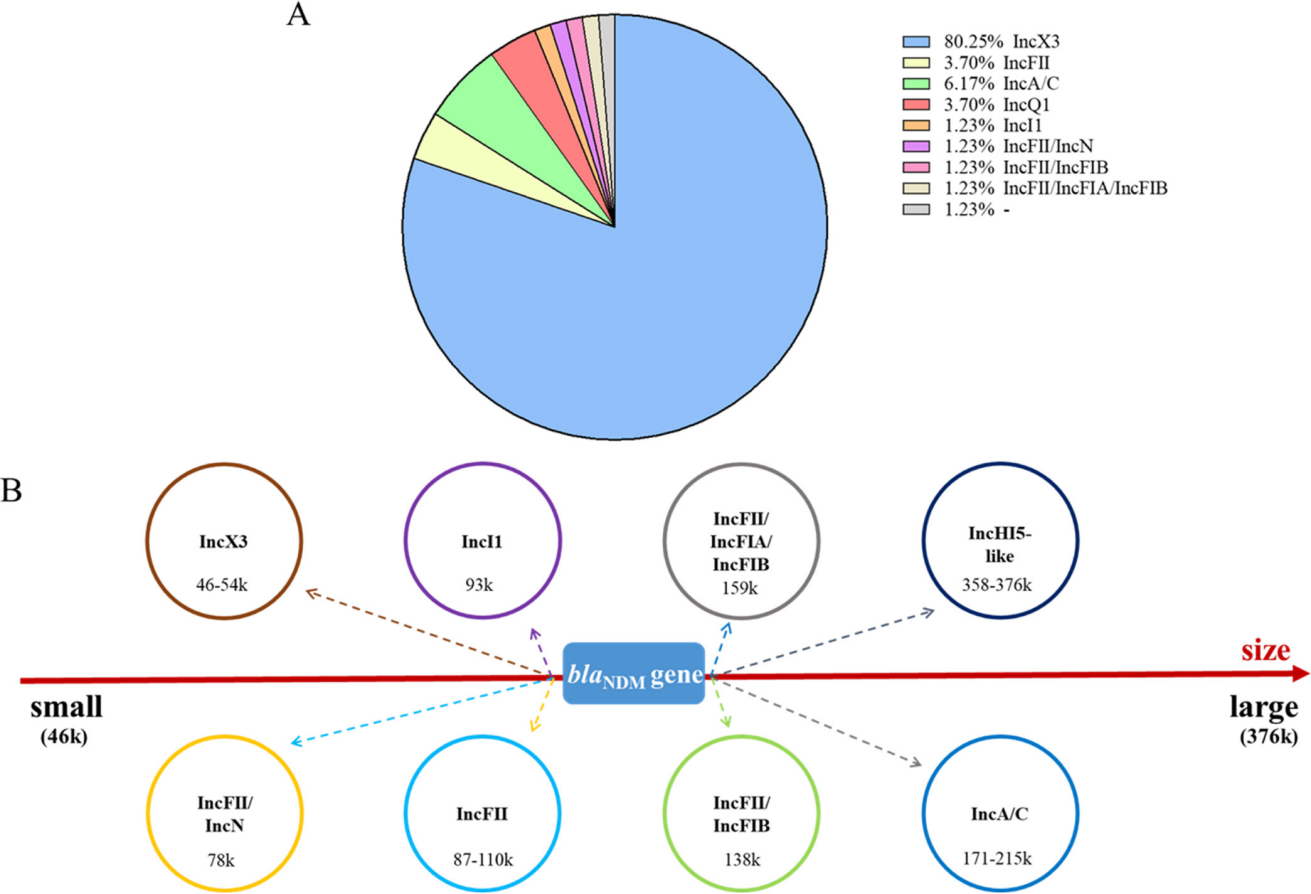
The distribution of different Inc group plasmids in all *bla*_NDM_-positive strains. (A) The percentage of Inc groups found in all *bla*_NDM_-positive strains. (B) Diversity of *bla*_NDM_-bearing plasmids in terms of replicon types and sizes. Eight different plasmids with various replicon combinations were identified, and each of them was labeled in different circle colors with plasmid types and sizes highlighted.

**FIG 4:**
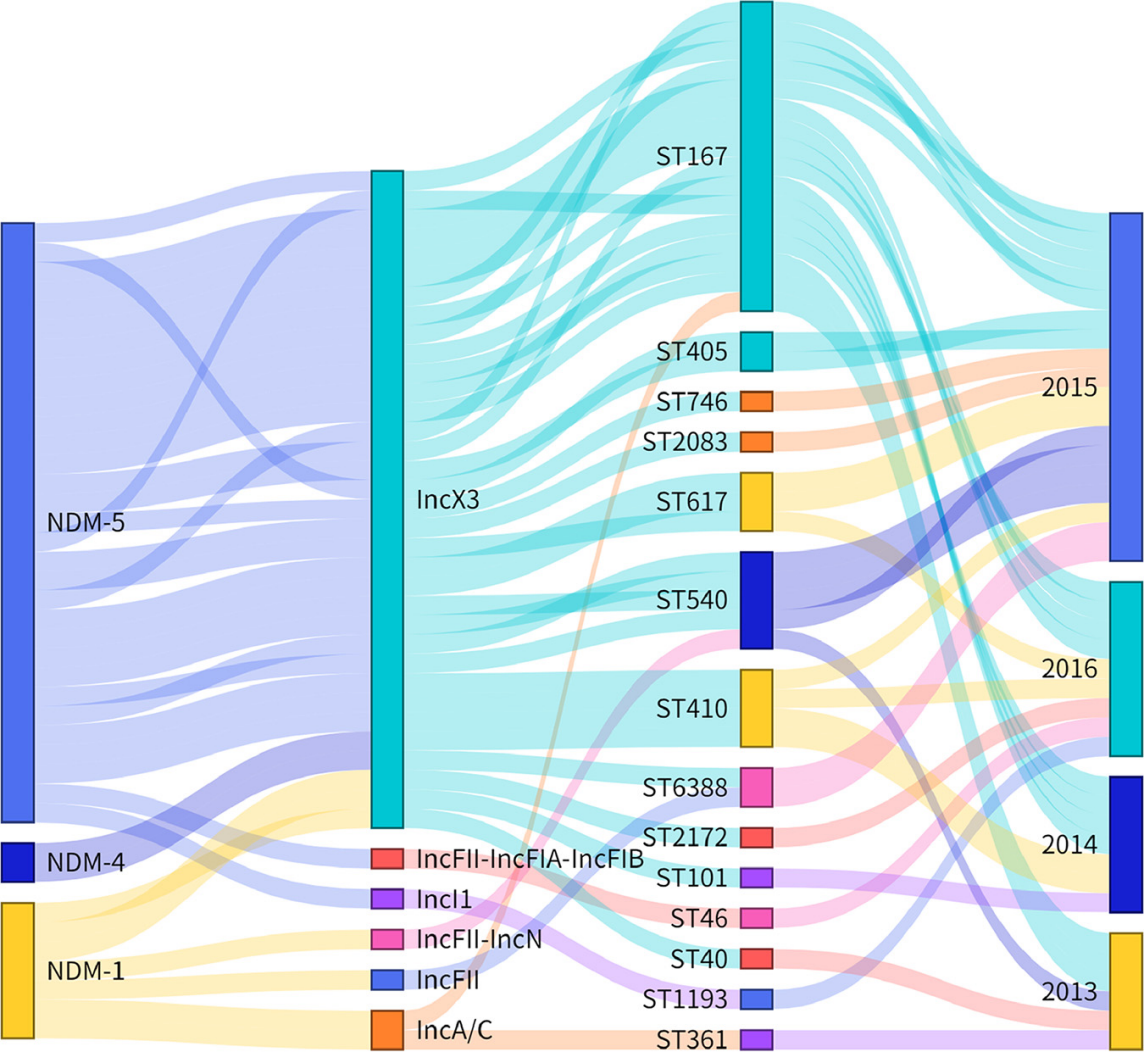
Sankey diagram combining different NDM subtypes, plasmid Inc types, ST types, and collection date. The diameter of the line is proportional to the number of isolates, which is also labeled at the consolidation points.

S1-PFGE and Southern blot analysis showed 77 *bla*_NDM_-positive strains located on plasmids. The Pr-15-2-50 was an exception, encoding a chromosomal *bla*_NDM_ gene, and four strains (KA-14-61, EC-14-2-92, EC-15-34, and EC-15-2-153) failed to produce a visible band; however, they were confirmed on plasmids during the transfer experiments and whole-genome sequencing (WGS) analysis. Notably, two different *bla*_NDM_-bearing plasmids, pECL-14-60-NDM-1-IncAC (IncC, 171,038 bp) and pECL-14-60-NDM-1 (IncX3, 53,023 bp), were identified in the strain ECL-14-60. These 81 *bla*_NDM_-harboring plasmids were categorized into nine different replicon types (Fig. 3A) with sizes ranging from ∼46 to ∼370 kb (Fig. 3B). The isolated Inc types of plasmids carrying *bla*_NDM_ genes were different each year; however, IncX3 *bla*_NDM_-positive plasmids were dominant through the period (Fig. 4 and Table 1). The bacteria carrying *bla*_NDM_-positive IncX3-type plasmids were diverse. Sixty-five NDM-producing IncX3 type plasmids with different sizes 54 kb and 46 kb (lacking the *bla*_SHV-12_-bearing segment) were found in 10 different bacterial species.

**FIG 5:**
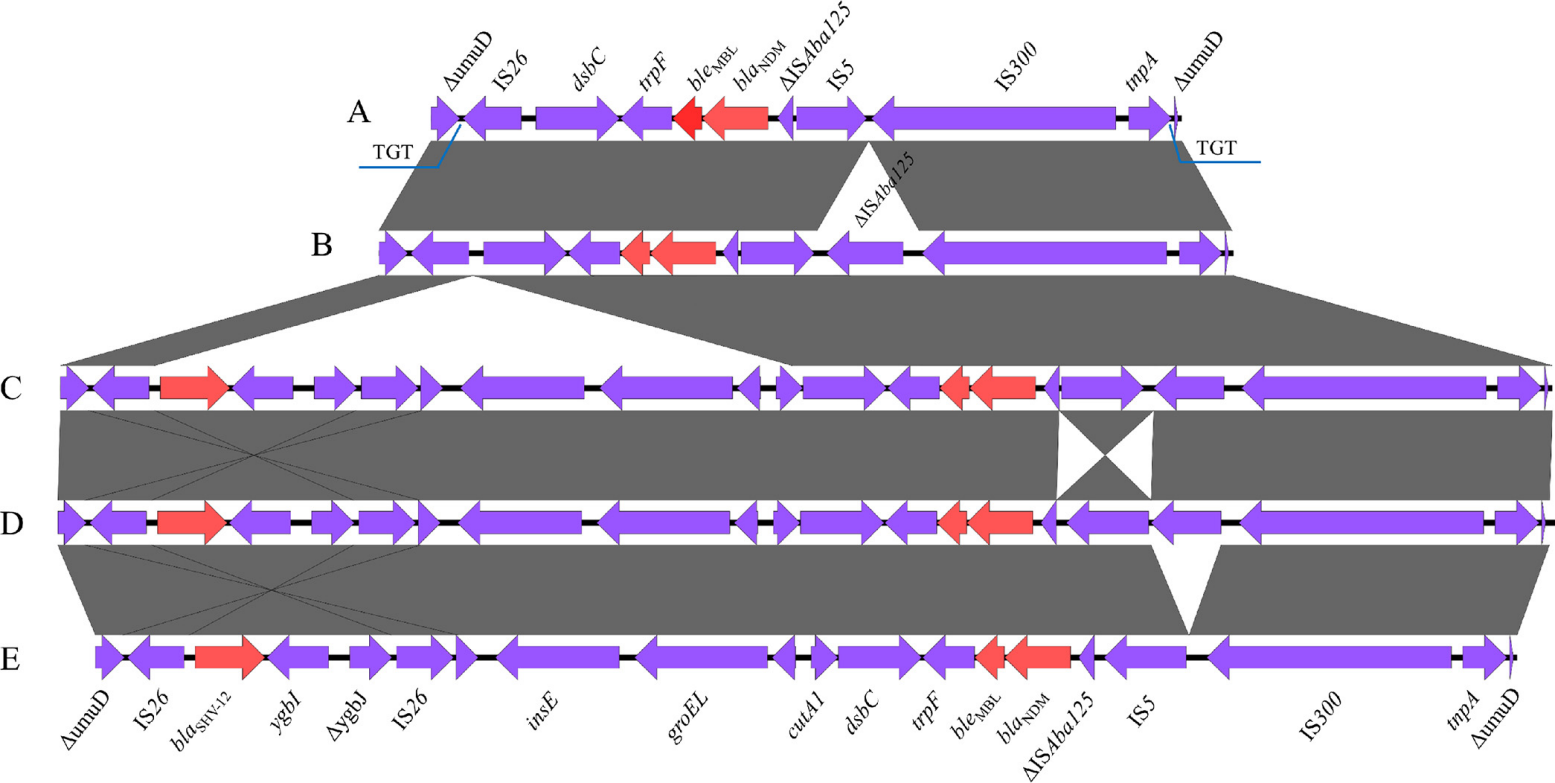
Different *bla*_NDM_ gene core genetic environments of the IncX3-type plasmids. A total of five (A–E) major types of *bla*_NDM_-bearing genetic contexts among the 42 *bla*_NDM_-bearing plasmids. Red arrows represent resistance genes.

In total, the environment around the *bla*_NDM_ gene located on the IncX3 plasmid can be classified into five major groups. These regions carrying the *bla*_NDM_ genes were all inserted into the *umuD* gene, and a 3-bp (TGT) direct repeat sequence formed at the insertion site. Group A (*n* = 1) is the simplest among several groups (Fig. 5). Compared with group A, group B (*n* = 29) had one more IS*Aba125* insertion downstream from the *bla*_NDM_. Group C (*n* = 20) had more 7,874 bp regions carrying the *bla*_SHV_ gene downstream from the IS*26* compared with group B. Group D had the reverse IS*5* arrangement compared with group C. Compared with group D, the region in group E lost the IS*Aba125* gene downstream from the *bla*_NDM_.

By connecting the *bla*_NDM_ subtypes of E. coli to plasmid types, ST types as well as the year of isolation (Fig. 4), we illustrated a complex combination of multiple genetic vehicles and diverse hosts in the spreading of the *bla*_NDM_ gene. Most of the *bla*_NDM-5_ genes were distributed on the IncX3 plasmids. Moreover, *bla*_NDM-1_ and *bla*_NDM-4_ were also found on the IncX3 plasmids. According to Fig. 4, IncX3 plasmids are the main *bla*_NDM_-positive plasmids isolated each year, and these plasmids are distributed in many different STs of E. coli. However, compared with other Inc-type NDM positive plasmids (Fig. S3), IncX3 type plasmids carried only a few antibiotic resistance genes, which may incur a low fitness cost to the host.

### Characterization of novel Inc-type and hybrid *bla*_NDM_-bearing plasmids.

**FIG 6:**
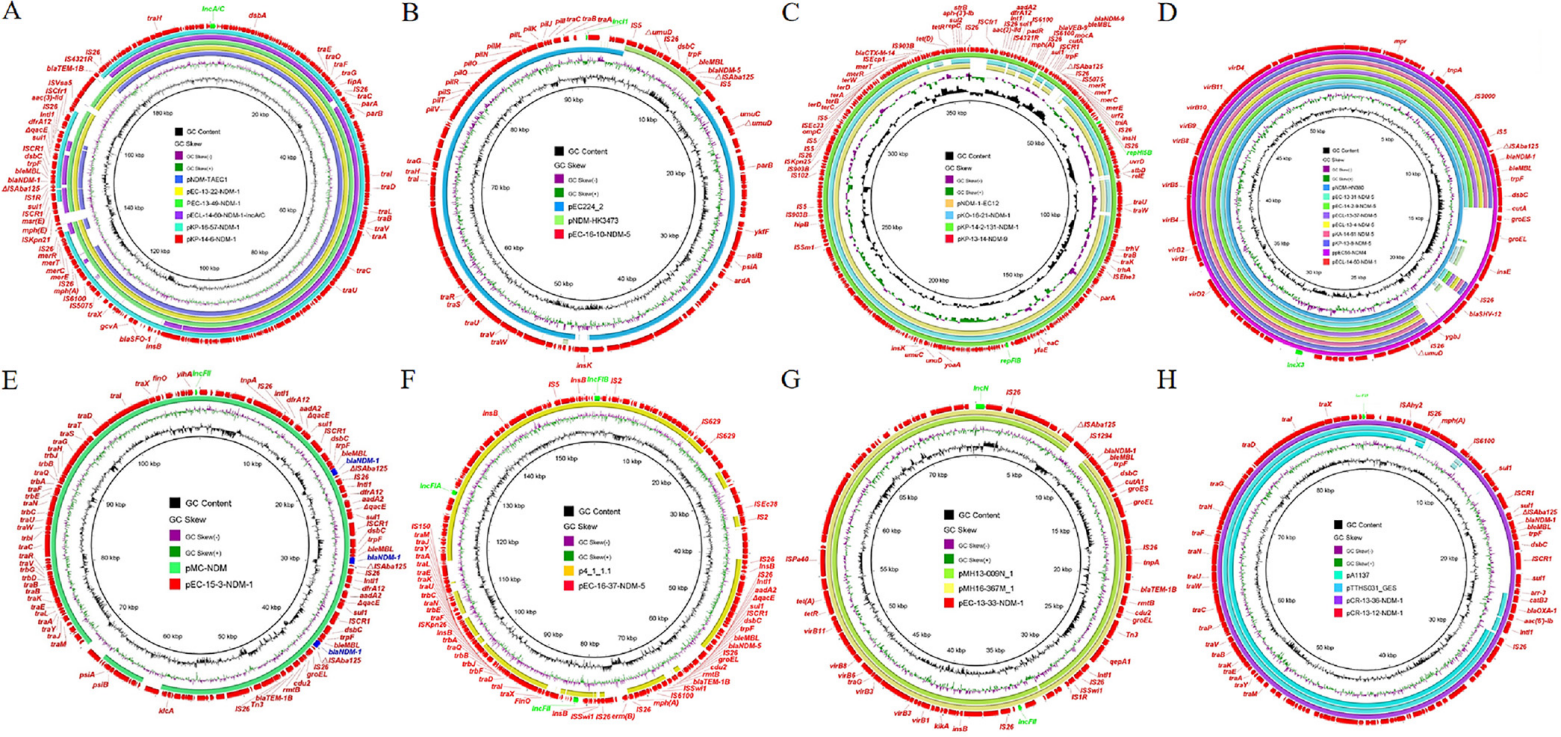
Circular comparison of different *bla*_NDM_-bearing plasmids with similar online plasmids. A–H represent different *bla*_NDM_-bearing plasmids with various replicons IncC, IncI1, IncHI5-like, IncX3, IncFII, IncFIA-IncFIB-IncFII, IncFII-IncN, and IncFII(p14).

In addition to IncX3 plasmids, other Inc-types of NDM-bearing plasmids were also detected in these strains (Fig. 6). To the best of our knowledge, the IncI1 plasmid pEC-16-10-NDM-5 characterized in this study is a novel *bla*_NDM_-bearing plasmid (Fig. S4 and Fig. 6). Plasmid pEC-16-10-NDM-5 was 92,260 bp in size and had an average G+C content of 50.6%. The BLAST comparison against the GenBank database showed that pEC-16-10-NDM-5 exhibited similarities to IncI1 plasmid pEC224_2 (CP018946). The main difference is that plasmid pEC-16-10-NDM-5 has an additional 8,698 bp complex transposon structure composed of two IS*5* and a *bla*_NDM-5_-bearing region (IS*5*-*hp*-*hp*-Δ*umuD*-IS*26*-*dsbC*-*trpF*-*ble*_MBL_-*bla*_NDM-5_-ΔIS*Aba125*-IS*5*). This additional transposon structure is similar to the IncX3 plasmid pNDM-HK3473 (MH234506) carrying the *bla*_NDM-5_ gene. It is flanked by 15 bp inverted repeats (TAGGGAAGGTGCGAA) on either side. This phenomenon indicates that the *bla*_NDM-5_ could be transferred through this complex transposon and integrated into the IncI1 plasmid (Fig. S5).

Five IncFII *bla*_NDM_-bearing plasmids were also identified among the 22 plasmids with complete and circular sequences using Nanopore sequencing (Table 2). The pMLST of the pEC-15-3-NDM-1 plasmid was F2:A-:B-, and the size of the plasmid is 109,944 bp. BLASTn analysis of the pEC-15-3-NDM-1 plasmid showed that it had 99% nucleotide identity at more than 95% coverage to pMC-NDM (HG003695). The main difference between them was the copy number of *bla*_NDM-1_. Three 10,461 bp region repeats were found on the pEC-15-3-NDM-1 plasmid, which carried a variety of resistance genes, including *bla*_NDM-1_, *dfrA12*, *aadA2*, *sul1*, and *ble*_MBL_. According to the result of BLASTn, two Y2:A-:B- (pCR-13-12-NDM-1 and pCR-13-36-NDM-1) plasmids of IncFII were similar to the pA1137 (NZ_MF190369) and pTTHS031_GES (NZ_LC589514) plasmids in the NCBI database. In contrast to the plasmids in this study, they all lacked regions carrying the *bla*_NDM_ gene, implying that the regions carrying the *bla*_NDM_ may insert progenitors before forming these plasmids. Moreover, two-hybrid plasmids pEC-16-37-NDM-5 (IncFII-IncFIA-IncFIB) and pEC-13-33-NDM-1 (IncFII-IncN) were also found. The pEC-16-37-NDM-5 plasmid was similar to the online IncFII-IncFIA-IncFIB plasmid p4_1_1.1 (NZ_CP023845) in E. coli. The plasmid pEC-13-33-NDM-1 was similar to both of the online IncFII-IncN plasmids pMH13-009N_1 (AP018566) and pMH16-367M_1 (AP018565) found in Proteus mirabilis and Morganella morganii, respectively. The core genetic environment of *bla*_NDM-5_ in pEC-16-37-NDM-5 is IS*CR1*-*dsbC*-*trpF*-*ble*_MBL_-*bla*_NDM-5_-ΔIS*Aba125*-IS*26*. Although there are only four IncFII-type plasmids carrying *bla*_NDM-1_, the gene environment around *bla*_NDM-1_ could be divided into three categories: Tn*As3*-*groEL*-*cutA*-*dsbC*-*trpF*-*ble*_MBL_-*bla*_NDM-1_-ΔIS*Aba125*-IS*1294* (pEC-13-33-NDM-1), IS*CR1*-*dsbC*-*trpF*-*ble*_MBL_-*bla*_NDM-1_-ΔIS*Aba125*-IS*26* (pEC-15-3-NDM-1 and pCR-13-12-NDM-1), and IS*CR1*-*dsbC*-*trpF*-*ble*_MBL_-*bla*_NDM-1_-ΔIS*Aba125*-IS*CR1* (pCR-13-36-NDM-1).

**TABLE 2:** Basic information of 22 *bla*_NDM_-bearing plasmids resolved by Illumina and Nanopore long-read sequencing

Plasmid	Strain	Status	Size (bp)	Inc-type	Assembly method	Sequencing technology	Accession no.	Resistance genes
pCR-13-12-NDM-1	CR-13-12	complete	86 619	IncFII	Unicycler	Oxford Nanopore MinION, Illumina	NZ_MN175388	*aac(6′)-Ib*, *arr-3*, *bla*_NDM-1_, *bla*_OXA-1_, *ble*_MBL_, *catB3*, *mph*(A), *sul1*
pCR-13-36-NDM-1	CR-13-36	complete	86 619	IncFII	Unicycler	Oxford Nanopore MinION, Illumina	MZ857202	*aac(6′)-Ib*, *arr-3*, *bla*_NDM-1_, *bla*_OXA-1_, *ble*_MBL_, *catB3*, *mph*(A), *sul1*
pEC-13-22-NDM-1	EC-13-22	complete	212 551	IncC	Unicycler	Oxford Nanopore MinION, Illumina	MZ836796	*aac(3)-IId*, *aph(3′)-VI*, *bla*_NDM-1_, *bla*_SFO-1_, *bla*_TEM-1_, *ble*_MBL_, *dfrA12*, *mph*(A), *mph*(E), *msr*(E), *sul1*
pEC-13-31-NDM-5	EC-13-31	complete	49 021	IncX3	Unicycler	Oxford Nanopore MinION, Illumina	MZ836797	*bla*_NDM-5_, *ble*_MBL_
pEC-13-33-NDM-1	EC-13-33	complete	74 978	IncFII-IncN	Unicycler	Oxford Nanopore MinION, Illumina	MZ836798	*bla*_NDM-1_, *bla*_TEM-1_, *ble*_MBL_, *qepA1*, *rmtB1*, *tet*(A)
pEC-13-49-NDM-1	EC-13-49	complete	214 323	IncC	Unicycler	Oxford Nanopore MinION, Illumina	MZ836799	*aac(3)-IId*, *aph(3′)-VI*, *bla*_NDM-1_, *bla*_SFO-1_, *bla*_TEM-1_, *ble*_MBL_, *dfrA12*, *mph*(A), *mph*(E), *msr*(E), *sul1*
pEC-14-2-9-NDM-5	EC-14-2-9	complete	46 161	IncX3	Unicycler	Oxford Nanopore MinION, Illumina	MZ836800	*bla*_NDM-5_, *ble*_MBL_
pEC-15-3-NDM-1	EC-15-3	complete	109 944	IncFII	Unicycler	Oxford Nanopore MinION, Illumina	NZ_MN061455	*aadA2*, *bla*_NDM-1_, *bla*_TEM-1_, *ble*_MBL_, *dfrA12*, *rmtB1*, *sul1*
pEC-16-10-NDM-5	EC-16-10	complete	92 260	IncI1	Unicycler	Oxford Nanopore MinION, Illumina	MZ836801	*bla*_NDM-5_, *ble*_MBL_
pEC-16-37-NDM-5	EC-16-37	complete	157 578	IncFII-IncFIA-IncFIB	Unicycler	Oxford Nanopore MinION, Illumina	MZ836802	*aadA2*, *bla*_NDM-5_, *bla*_TEM-1_, *ble*_MBL_, *dfrA12*, *erm*(B), *mph*(A), *rmtB1*, *sul1*
pEC55-NDM4	EC-14-55	complete	54 035	IncX3	Unicycler	Oxford Nanopore MinION, Illumina	NZ_KX470734	*bla*_NDM-4_, *bla*_SHV-12_, *ble*_MBL_
pECL-13-37-NDM-5	ECL-13-37	complete	46 161	IncX3	Unicycler	Oxford Nanopore MinION, Illumina	MZ836804	*bla*_NDM-5_, *ble*_MBL_
pECL-13-4-NDM-5	ECL-13-4	complete	46 161	IncX3	Unicycler	Oxford Nanopore MinION, Illumina	MZ836803	*bla*_NDM-5_, *ble*_MBL_
pECL-14-60-NDM-1-IncAC	ECL-14-60	complete	171 038	IncC	Unicycler	Oxford Nanopore MinION, Illumina	MZ836805	*aac(6′)-Ib*, *aph(3′)-Ia*, *armA*, *arr-3*, *bla*_NDM-1_, *bla*_OXA-1_, *ble*_MBL_, *catB3*, *mph*(E), *msr*(E), *qnrA7*, *sul1*, *sul2*
pECL-14-60-NDM-1	ECL-14-60	complete	53 023	IncX3	Unicycler	Oxford Nanopore MinION, Illumina	NZ_MN061454	*bla*_NDM-1_, *bla*_SHV-12_, *ble*_MBL_
pKA-14-61-NDM-5	KA-14-61	complete	46 161	IncX3	Unicycler	Oxford Nanopore MinION, Illumina	MZ836806	*bla*_NDM-5_, *ble*_MBL_
pKO-16-21-NDM-1	KO-16-21	complete	376 570	IncHI5-like	Unicycler	Oxford Nanopore MinION, Illumina	MZ836807	*aac(3)-IId*, *aadA16*, *aph(3′')-Ib*, *aph(6)-Id*, *arr-3*, *bla*_NDM-1_, *bla*_TEM-1_, *ble*_MBL_, *dfrA27*, *mph*(A), *qnrB6*, *sul1*, *sul2*
pKP-13-14-NDM-9	KP-13-14	complete	358 655	IncHI5-like	Unicycler	Oxford Nanopore MinION, Illumina	NZ_MN175386	*aac(3)-IId*, *aadA2*, *aph(3′')-Ib*, *aph(6)-Id*, *bla*_CTX-M-14_, *bla*_NDM-9_, *bla*_TEM-1_, *ble*_MBL_, *dfrA12*, *mph*(A), *tet*(D), *sul1*, *sul2*
pKP-13-8-NDM-5	KP-13-8	complete	46 161	IncX3	Unicycler	Oxford Nanopore MinION, Illumina	NZ_MN175389	*bla*_NDM-5_, *ble*_MBL_
pKP-14-2-131-NDM-1	KP-14-2-131	complete	358 158	IncHI5-like	Unicycler	Oxford Nanopore MinION, Illumina	MZ836808	*aac(3)-IId*, *aadA2*, *aph(3′')-Ib*, *aph(6)-Id*, *bla*_CTX-M-14_, *bla*_NDM-1_, *bla*_TEM-235_, *ble*_MBL_, *dfrA12*, *mph*(A), *tet*(D), *sul1*, *sul2*
pKP-14-6-NDM-1	KP-14-6	complete	199 120	IncC	Unicycler	Oxford Nanopore MinION, Illumina	NZ_MN175387	*aac(3)-IId*, *bla*_NDM-1_, *bla*_SFO-1_, *bla*_TEM-1_, *ble*_MBL_, *dfrA12*, *mph*(A), *mph*(E), *msr*(E), *sul1*
pKP-16-57-NDM-1	KP-16-57	complete	180 309	IncC	Unicycler	Oxford Nanopore MinION, Illumina	MZ836809	*aac(3)-IId*, *aadA2*, *bla*_NDM-1_, *bla*_SFO-1_, *ble*_MBL_, *dfrA12*, *mph*(A), *sul1*

The characteristics of the five NDM-positive plasmids (171 kb–215 kb) of the IncC type were also analyzed (Fig. 6). The *bla*_NDM_ subtypes carried by these plasmids were all *bla*_NDM-1_, and most of them shared similar backbones. Despite their similar backbones, there are three types of genetic environments around *bla*_NDM-1_: IS*CR1*-*dsbC*-*trpF*-*ble*_MBL_-*bla*_NDM-1_-ΔIS*Aba125*-IS*1R* (pECL-14-60-NDM-1 and pKP-14-6-NDM-1), IS*CR1*-*dsbC*-*trpF*-*ble*_MBL_-*bla*_NDM-1_-IS*Aba125* (pEC-13-22-NDM-1 and pEC-13-49-NDM-1), and IS*CR1*-*dsbC*-*trpF*-*ble*_MBL_-*bla*_NDM-1_-ΔIS*Aba125*-IS*26* (pKP-16-57-NDM-1). Among these five IncC plasmids, pEC-13-22-NDM-1 and pEC-13-49-NDM-1 were isolated from E. coli, pKP-16-57-NDM-1 and pKP-14-6-NDM-1 from K. pneumoniae, and pECL-14-60-NDM-1 from E. hormaechei. BLASTn comparison with the NCBI database showed similarities to pNDM-TAEC1 (NZ_MH001166) found in E. coli.

It is worth noting that three plasmids belonged to the recently discovered IncHI5-like plasmids (Fig. 6). The three *bla*_NDM_-harboring IncHI5-like plasmids ranged from 358 to 376 kb and possessed the same plasmid backbone structure. The BLAST comparison against the GenBank database showed that plasmid pKO-16-21-NDM-1 from K. oxytoca exhibited similarities to the same Inc-type plasmids pKP19-3023-374k (CP063748) and pKP19-3088-375k (CP063149), which were collected from K. pneumoniae. The core genetic environment of *bla*_NDM_ (IS*CR1*-*sul1*-Δ*qacE*-*bla*_NDM-1_-*ble*_MBL_-*trpF*-*dsbC*-IS*CR1*) carried on the plasmid pKO-16-21-NDM-1 was similar to the pKP19-3023-374k plasmid. This is the first time that a *bla*_NDM_-positive IncHI5-like plasmid has been found in K. oxytoca. The pKP-13-14-NDM-9 plasmid that was isolated from K. pneumoniae was 358,655 bp in size. Although IncHI5-like plasmids were reported to carry *bla*_NDM-1_ in previous studies (12, 13), pKP-13-14-NDM-9 was the first IncHI5-like plasmid positive for *bla*_NDM-9_. The core genetic environment of *bla*_NDM-9_ is IS*26*-ΔIS*Aba125*-*bla*_NDM-9_-*ble*_MBL_-*trpF*-*mocA*-*cutA*-IS*CR1*, and a similar genetic environment (IS*26*-ΔIS*Aba125*-*bla*_NDM-1_-*ble*_MBL_-*trpF*-*mocA*-*cutA*-IS*CR1*) was found in pKP-14-2-131-NDM-1.

Four of the 81 strains were found to carry both *bla*_NDM_ and *mcr* genes (*mcr-1*, *n* = 1, *mcr-9*, *n* = 3). The *mcr-1* gene was located on a 60,961 bp plasmid designated as pEC-15-3-mcr-1 in the incompatibility group IncI2 (Fig. S6). The plasmids similar to pEC-15-3-mcr-1 in the public database were the E. coli plasmid pAH62-1 (NZ_CP055260) and Salmonella plasmid pS304_2 (NZ_CP061128), which showed 100% coverage and identity. Moreover, three strains were found (CF-15-2-29, ECL-16-5, and ECL-16-79) carrying the *mcr-9*. Online BLAST (Fig. S7) showed that *mcr-9*-positive plasmids all belonged to IncHI2A-IncHI2 and showed similarities to the pBSI034-MCR9 (NZ_MN937241) plasmid. Strains carrying *mcr-9* were usually resistant to polymyxin; however, ECL-16-79 was susceptible to polymyxin. It has been reported that the deletion of the two-component system *qseCB* may silence the *mcr-9* gene (14). However, the ECL-16-79 strain contains the two-component system *qseCB*, and other genes or molecules may regulate the expression of *mcr-9*. Further investigations are needed to decipher the underlying molecular mechanisms.

### Two tandem copies of *bla*_NDM-1_ in the chromosome.

In addition to the plasmid-mediated *bla*_NDM_ genes, we also found the *bla*_NDM-1_ on the chromosome of the *P. rettgeri* strain Pr-15-2-50. The size of the genome was 4,648,900 bp, with 40.3% GC content. Two copies of *bla*_NDM-1_ were found on the chromosome of the Pr-15-2-50. On comparing the Pr-15-2-50 chromosome with FZB001 (CP060821) and AR0156 (CP021852), we found a 40,775 bp Tn*7*-like transposon structure carrying the *bla*_NDM_ gene, inserted into the chromosomal region (Fig. S8). This Tn*7*-like transposon had an average GC content of 48.8% and similarly to the p2BJAB07104 (CP003907) plasmid, it was surrounded by 11 bp inverted repeats (ACAAAATAGAT), implying that the transposon could translocate between chromosomes and plasmids. However, this plasmid lacked the *bla*_NDM_-bearing region. The 5,250 bp *bla*_NDM_-carrying region (IS*CR1*-*dsbC*-*trpF*-*ble*_MBL_-*bla*_NDM-1_-ΔIS*Aba125*-Δ*sul1*) may be incorporated because of the IS*CR1*-mediated insertion, similar to previous reports(15). Moreover, a 4,390 bp integron (*intI2*-*lnu*(F)-*dfrA1*-*aadA1*-Δ*qacE*-*sul1*) was found downstream to the *bla*_NDM-1_ gene. Despite these reports, IS*CR1*-mediated copies of *bla*_NDM_ have been found on these chromosomes. However, the IS*CR1*-mediated transposable units on P. aeruginosa MMA83 (IS*CR1*-*aph(3′)-VIa*-IS*Aba125*-*bla*_NDM-1_-*sul1*), E. coli Y5 (IS*CR1*-*traF*-*ble*_MBL_-*bla*_NDM-1_-ΔIS*Aba125*-*catB3*-*arr-3*-Δ*qacE*-*sul1*), and P. mirabilis XH1653 (*sul1*-*arr-3*-*cat*-*bla*_NDM-1_-*bleo*-IS*CR1*) are different from the Pr-15-2-50 (IS*CR1*-*dsbC*-*trpF*-*ble*_MBL_-*bla*_NDM-1_-ΔIS*Aba125*-Δ*sul1*) strain in this study (15–17). The *ble* and *sul1* genes were also detected in these transposable units. This suggests that *bla*_NDM_ may cotransfer with other resistance genes.

## DISCUSSION

*bla*_NDM-1_ was discovered in 2009. Since then, CRE strains carrying *bla*_NDM-1_ and its variants have spread in more than 55 countries worldwide. Asian countries such as India, Pakistan, and China are considered major reservoirs of *bla*_NDM_ (6). The *bla*_NDM-1_-positive strains were first isolated in clinical stool samples in China in 2010, followed by an increasing number of *bla*_NDM_-positive strains. In 2013, 17 *bla*_NDM_-positive strains (38.64%) were obtained from 44 CRE strains isolated from hospitals in Henan, which was an increase compared to 2011–2012 (7). However, the positive rate decreased to 18.89% (17/90) and 17.13% (31/181) in 2014 and 2015, respectively. This may be the result of the effective clinical infection control measures. However, there was an increase in 2016, with the isolation rates reaching up to 21.79% (17/78).

ST11 is the most common type of *bla*_NDM_-positive K. pneumoniae that was reported (18, 19). Moreover, ST11 K. pneumoniae often had hypervirulent and/or multidrug resistant phenotypes (20). However, only one strain of ST11 K. pneumoniae was found in this study. The ST types of K. pneumoniae were more diverse, indicating that K. pneumoniae carrying *bla*_NDM_ in Henan is not clonally transferred. Moreover, we found diverse E. coli STs, and ST167 was predominant among them. This phenomenon is similar to previous domestic reports (21, 22). ST167 NDM-producing E. coli strains are not only widely disseminated in China (11, 23); they also cause infections worldwide (6, 24, 25), which has gained much attention. Consistent with this study, the *bla*_NDM-5_ gene is mainly carried by E. coli of ST167 (26, 27), suggesting that ST167 E. coli is an important repository of *bla*_NDM-5_. More importantly, ST167 *bla*_NDM_-positive E. coli strains have been found in companion animals (28, 29), which suggests that the ST167 E. coli carrying *bla*_NDM-5_ gene could be transmitted between animals and humans.

Four NDM subtypes (NDM-1, NDM-4, NDM-5, and NDM-9) were found in 81 NDM-producing strains; however, from 2011 to 2012, all *bla*_NDM_-positive strains isolated from Henan were *bla*_NDM-1_ (7). Since the isolation of *bla*_NDM-5_ in Henan in 2013, the detection rate has gradually increased. It has now become the main subtype of *bla*_NDM_. The *bla*_NDM-1_ detection rates have been decreasing each year; however, it remains the main epidemic subtype. Previous studies have shown that NDM-5 exhibits higher hydrolytic activity toward carbapenems and cephalosporins compared with NDM-1 (30). This may be caused by the increase in the usage of carbapenems in clinical treatment. It has been shown that IncX3 plasmids could promote the transmission of NDM-5, and the plasmids carrying NDM-5 demonstrated high stability (31, 32). In this study, most of the *bla*_NDM-5_ genes were carried by IncX3 plasmids, which led to a higher prevalence. The increasing prevalence of *bla*_NDM-5_-positive strains should be of high concern.

Carbapenem and colistin are considered the last line of defense in the treatment of severe infections caused by extensively drug-resistant bacteria. Only a few articles have previously reported the coexistence of *bla*_NDM-1_ and *mcr-9* genes (33, 34). Four strains with the coexistence of *bla*_NDM_ and *mcr* genes were found in this study. This phenomenon greatly increases the risk of treatment failure. The *bla*_IMP-4_-producing *Enterobacterales* have been reported sporadically in China (11, 35). Only two strains harboring the *bla*_IMP-4_ gene were found among the 81 *bla*_NDM_-positive strains. Moreover, multiple resistance genes are often present on plasmids carrying *bla*_NDM_ genes, which greatly increases the risk of cotransmission of multiple resistance genes.

Except for the strain Pr-15-2-50, the *bla*_NDM_ gene was located on the plasmids, which might be the main mode of *bla*_NDM_ transmission. A variety of *bla*_NDM_-positive plasmids with different Inc types and sizes were found in the 80 strains, mainly IncX3 type, which is similar to previous reports. In this study, the ST type of NDM-producing strains carrying the IncX3 plasmid was mainly ST167. This highly prevalent ST and plasmid type promotes the transmission of *bla*_NDM_ further and seriously threatens public health. We also discovered a novel *bla*_NDM_-bearing plasmid pEC-16-10-NDM-5 (IncI1). IncI1 plasmid belongs to the narrow-host range plasmid type (36) and was only found in *Enterobacterales*. Several articles have pointed out that IncI1 plasmids frequently carry genes encoding antibiotic resistance, especially the extended-spectrum beta-lactamase genes (37–39). These plasmids are widely distributed in animals and patients worldwide (40, 41). The IncHI plasmid has a wide host range and plays an important role in the transmission of resistance genes (42, 43). Previously, it was shown that a variety of carbapenemase genes were found on the IncHI5 plasmids (44), which poses a great threat to clinical treatment. The two IncHI5-like plasmids, carrying both carbapenem and tigecycline resistance genes, were found in our recent study (12), severely restricting the clinical treatment options. In this study, the new *bla*_NDM_ core genetic environment was found in the IncHI5-like plasmids, suggesting that this plasmid has evolved as a novel MDR plasmid and needs to be continuously monitored.

### Conclusion.

To date, NDM is the predominant mechanism for CRE in humans. Carbapenem, polymyxin, and tigecycline are regarded as the last line of defense in the clinical treatment of MDR infections. In recent years, several studies have found that *bla*_NDM_ coexists with mobile colistin (*mcr*) and tigecycline resistance genes (*tet*(X) and *tmexCD-toprJ*), making clinical treatment extremely difficult. Therefore, continuous long-term surveillance for pathogens that clinically harbor *bla*_NDM_ is important. This study conducted an in-depth analysis of *bla*_NDM_-positive clinical strains and confirmed that the vast majority of *bla*_NDM_ genes were distributed on plasmids of different Inc types, and are transmitted by horizontal transfer of plasmids. The emergence of *Enterobacterales* carrying both *bla*_NDM_ and other resistance genes, such as *mcr*, is worrying. These isolates can seriously limit clinical treatment options. Therefore, there is an urgent need for large-scale monitoring and the development of effective control measures.

## MATERIALS AND METHODS

### Bacterial isolates.

The samples in this study were obtained between 2013 and 2016 at an affiliated hospital of Zhengzhou University. This study did not exclude patients based on age, gender, or symptoms. Moreover, the samples collected were nonduplicate isolates from different patients. CRE was defined as *Enterobacterales* resistant to at least one carbapenem (meropenem or imipenem). A total of 391 CRE strains were collected from blood, urine, sputum, wound, tissue, pus, swab, drainage liquid, secreta, bile, ascites, sanies, joint fluid, and urine tube tips. Clinical data of each patient were collected from the clinical and medical record system. Extracted clinical information included the date of collection, patient age, sex, source of isolate, ward type, and outcome (alive or dead). The *bla*_NDM_-positive strains were screened and confirmed using PCR and Sanger sequencing, respectively. All *bla*_NDM_-positive isolates were sent to Zhengzhou University for subsequent experiments. This study was approved by the Ethics Committee of Zhengzhou University with a waiver of informed consent because of the retrospective nature of the study.

### PCR screening and antimicrobial susceptibility testing.

The presence of carbapenem resistance genes (*bla*_NDM_, *bla*_IMP_, *bla*_KPC_, *bla*_VIM,_ and *bla*_OXA-48_) and other important resistance genes (*mcr-1*, *bla*_SHV_, and *bla*_TEM_) was investigated using PCR with the primers (Table S1). The PCR amplified products were confirmed using gel electrophoresis and Sanger sequencing. All CRE species identification was carried out by the automated Vitek 2 system. Antimicrobial susceptibility testing of clinical strains was performed against 17 antimicrobials by determining the MICs using the broth microdilution method, and E. coli ATCC 25922 was used as the quality control. All antibiotic breakpoints were interpreted according to CLSI guidelines (45); however, tigecycline (>2 mg/L) was interpreted according to the EUCAST criteria.

### Conjugation, S1-PFGE, and Southern blot.

The conjugation experiment was performed with each of the *bla*_NDM_-positive strains using a rifampicin-resistant E. coli EC600 or sodium azide-resistant E. coli J53 recipients. The donor and recipient were mixed in a ratio of 1:1 and incubated statically in an LB broth at 35°C for 24 h. Transconjugants on the LB agar plates containing double antibiotics (meropenem 2 mg/L and rifampicin 100 mg/L, or meropenem 2 mg/L and sodium azide 200 mg/L) were selected and confirmed using PCR and PFGE, respectively. Transfer frequencies were calculated as the number of transconjugants/total number of recipients.

S1-PFGE and Southern blot analyses were performed to determine the plasmid sizes and genomic positions of *bla*_NDM_. To elucidate the genetic environments of *bla*_NDM_ genes, 22 representative *bla*_NDM_-carrying plasmids were selected based on the plasmid replicon types and sizes to perform Nanopore sequencing to obtain the complete plasmid sequences.

### WGS procedures and analyses.

We characterized the genetic features and resistomes of the *bla*_NDM_-positive CRE. The genomes of all *bla*_NDM_-positive strains were extracted with the FastPure bacterial DNA isolation minikit (catalog no. DC103; Vazyme) and evaluated using 1% (wt/vol) agarose gel electrophoresis. The concentration and purity were quantified using the Qubit 4 Fluorometer and Nanodrop. The genomic DNA samples were sequenced using the Illumina Hiseq 2500 platform generating 2 × 150 bp paired-end reads. Twenty-two representative strains were sequenced with the Nanopore long-read sequencing platform according to resistant phenotypes and genotypes (46). The Rapid Barcoding Kit RBK004 was used to construct the long-read sequencing libraries, which were subjected to Nanopore sequencing in MinION R9.4.1 flow cells.

The Illumina paired-end reads were *de novo* assembled using the SPAdes version 3.14.0, and contigs less than 200 bp in length were removed (47). Unicycler v. 0.4.8 was used for hybrid assembly of genomes with the combination of Illumina short reads and Nanopore long reads with default parameters (48). For intricate regions that could not be resolved using the hybrid assembly method, Nanopore sequencing data were assembled using the long-read assembler Flye v. 2.4.2 to acquire accurate structures of complex genomic regions (49). The genomes were annotated using the online tool RAST (http://rast.nmpdr.org/). ResFinder and PlasmidFinder (http://cge.cbs.dtu.dk/services/) were used to identify antimicrobial resistance genes and plasmid replicon types with default parameters. The virulence factors in the assembled genome sequences were identified using the Kleborate software (50) and the virulence factor database (last updated 14th October 2020) in abricate v.1.0.1 (https://github.com/tseemann/abricate) with default parameters. Multilocus sequence typing (MLST) of the 81 *bla*_NDM_-positive isolates was conducted using mlst (https://github.com/tseemann/mlst). The plasmid comparison maps were constructed and displayed by using BRIG v. 0.95 and Easyfig v. 2.2.3 (51, 52), respectively. The core genes in the genomes of *bla*_NDM_-positive CRE were identified using Roary (53). The phylogenetic trees of *bla*_NDM_-positive strains were constructed using FastTree (54) based on the core single-nucleotide polymorphism (SNP) alignments with default parameter settings and visualized using iTOL (https://itol.embl.de).

### Data availability.

The sequence data generated in this study have been submitted to the NCBI BioProject database (https://www.ncbi.nlm.nih.gov/bioproject/) under accession numbers PRJNA752009, and individual accession numbers of 22 *bla*_NDM_-bearing plasmids are listed in Table 2.
