# Supplementary material for: Genomic Epidemiology Insights on NDM-Producing Pathogens Revealed the Pivotal Role of Plasmids on blaNDM Transmission
Source: Microbiol Spectr. 2022 Feb 28;10(2):e02156-21. doi: 10.1128/spectrum.02156-21 (PMC9049954; doi:10.1128/spectrum.02156-21)
Supplement: SUPPLEMENTAL FILE 1 — Supplemental material. Download SPECTRUM02156-21_Supp_1_seq11.pdf, PDF file, 1.3 MB [file spectrum02156-21_supp_1_seq11.pdf]

Supplementary Table 1. Primer sequences used in this study.

Primers	Primer sequences	Products (bp)	Genes
NDM-F	GCAGCTTGTCGGCCATGCGGGC	782	bla_{NDM}
NDM-R	GGTCGCGAAGCTGAGCACCGCAT		
IMP-F	GAAGGCGTTTATGTTCATAC	587	bla_{IMP}
IMP-R	GTACGTTTCAAGAGTGATGC		
KPC-F	TGGAAACTCCCCTACCTCTT	900	bla_{KPC}
KPC-R	CCACACTGATGCGTTCGTAT		
VIM-F	CGCGGTCGCCATCTTCACG	389	bla_{VIM}
VIM-R	TGCCCGAGCCAGTACCGCCAATG		
OXA-F	GGCAACCTCGTCGCCTTTA	438	bla_{OXA}
OXA-R	GCAAGTCGGCGTGTTTTCG		
MCR-1-F	ATCAGCCAAACCTATCCCATCG	564	*mcr-1*
MCR-1-R	GCAGACGCACAGCAATGCCTAT		
SHV-F	ATCGTGGAGTGATCGACATT	1565	bla_{SHV}
SHV-R	ATCGTGGAGTGATCGACATT		
TEM-F	ATAAAATTCTTGAAGAC	1075	bla_{TEM}
TEM-R	TTACCAATGCTTAATCA		

Supplementary Table 2. Antimicrobial susceptibility testing (MICs, mg/L) of 81 *bla*$_{NDM}$-positive strains.

Isolates	MEM	IPM	ATM	CAZ	CHL	CIP	CST	FOS	GEN	AMK	AMP	TET	TGC	CFX	CFZ	TZP	SXT
EC-13-1	16	4	64	>256	256	8	≤0.5	8	32	16	>256	256	1	>256	>256	>256	>32
ECL-13-2	64	32	64	>128	>128	16	2	32	>128	>128	>256	16	1	>256	>256	512	> 32
ECL-13-4	32	32	>128	>128	32	>32	1	≤1	1	16	>256	16	4	>256	>256	>1024	> 32
KP-13-7	8	32	>128	>128	>256	>32	2	16	>128	>128	>256	64	8	>256	>256	>1024	> 32
KP-13-8	8	64	>128	>128	>256	>32	0.5	16	128	4	>256	32	2	32	>256	1024	> 32
KP-13-11	8	64	>128	>128	64	0.5	32	16	1	8	>256	32	2	>256	>256	1024	> 32
CR-13-12	16	64	>128	>128	>256	1	8	16	256	2	>256	64	1	>256	>256	>1024	> 32
KP-13-14	16	64	>128	>128	8	8	1	16	32	2	>256	64	1	>256	>256	1024	> 32
EC-13-22	16	32	>128	>128	8	32	2	≤1	128	2	>256	64	0.5	>256	>256	>1024	> 32
EC-13-30	32	32	>128	>128	256	16	1	512	128	4	>256	4	0.25	>256	>256	>1024	> 32
EC-13-31	16	32	>64	>128	>128	16	0.5	512	128	4	>256	64	1	>256	>256	>512	> 32
EC-13-33	4	16	>128	>128	16	32	2	≤1	2	>128	>256	32	0.25	>256	>256	1024	> 32
CF-13-34	8	16	>128	>128	16	8	2	≤1	128	2	>256	>128	0.5	>256	>256	512	> 32
CR-13-36	4	64	128	>128	8	≤0.12	2	4	1	1	>256	8	1	>256	>256	512	> 32
ECL-13-37	32	64	>128	>128	16	32	1	32	>256	>128	>256	64	1	>256	>256	1024	> 32
EC-13-49	16	8	>128	>128	16	32	1	≤1	128	>128	>256	4	0.5	>256	>256	>1024	> 32
PM58	16	64	>128	>128	>256	16	>128	>1024	>256	>128	>256	128	8	>256	>256	32	> 32
KP-14-6	64	32	2	≤0.25	16	≤0.12	2	16	64	2	>256	64	0.5	>256	>256	>1024	0.5
CF-14-50	8	16	128	>128	>256	16	0.5	≤1	64	1	>256	8	1	>256	>256	1024	> 32
EC-14-54	64	64	64	64	>64	>64	2	≤1	64	8	>256	>64	2	>256	>256	>1024	> 32
EC-14-55	32	32	>64	>32	4	16	0.5	≤1	>32	<2	>256	>32	0.5	>256	>256	>1024	>32
ECL-14-56	8	32	128	>128	256	32	2	4	64	2	>256	8	1	>256	>256	512	> 32
ECL-14-58	16	32	128	>128	16	2	4	32	<0.5	2	>256	16	4	>256	>256	>1024	> 32
ECL-14-60	16	64	2	>128	16	16	2	32	>125	>256	>256	16	4	>256	>256	1024	1.5
KA-14-61	16	16	32	>128	8	0.25	1	4	64	2	>256	16	0.5	>256	>256	1024	> 32
KO-14-71	32	32	64	128	4	0.5	1	256	128	4	>256	32	1	>256	>256	512	> 32
KOR-14-72	32	0.5	32	8	>256	8	1	16	>256	>256	>256	32	0.5	>256	>256	1024	> 32
EC-14-2-9	64	64	64	64	>64	>64	2	≤1	>128	>64	>256	>64	0.25	>256	>256	>1024	> 32
EC-14-2-77	64	64	128	>128	4	16	1	≤1	>128	>128	>256	32	1	>256	>256	1024	> 32

EC-14-2-92	16	64	>64	>128	>128	8	1	1024	>128	>128	>256	32	0.25	>256	>256	>1024	> 32
EC-14-2-94	16	32	64	>128	>128	8	2	>1024	2	2	>256	32	≤0.25	>256	>256	>1024	> 32
KP-14-2-131	4	32	32	>128	>256	0.5	4	16	128	2	>256	4	1	>256	>256	>1024	> 32
EC-14-2-134	16	32	>64	>128	16	8	0.5	4	>128	>128	>256	8	≤0.25	>256	>256	>1024	>32
EC-15-3	64	128	128	128	>256	64	1	1024	>512	>256	>256	64	2	>256	>256	>512	>32
EC-15-10	16	64	64	>128	4	≤0.12	≤0.5	4	1	2	>256	2	≤0.25	>256	>256	>512	>32
CF-15-33	8	32	64	>128	32	0.25	1	≤1	1	2	>256	8	1	>256	>256	>512	<20
EC-15-34	64	128	64	>128	>128	8	≤0.5	>1024	>128	>128	>256	>128	0.5	>256	>256	1024	>32
KP-15-35	4	32	64	>128	4	0.12	4	8	2	1	>256	4	1	>256	>256	>512	>32
CF-15-43	4	16	128	>128	8	16	1	≤1	256	8	>256	4	1	>256	>256	>512	> 32
CF-15-61	8	16	64	>128	>256	16	0.5	≤1	64	1	>256	8	0.5	>256	>256	512	>32
EC-15-2-1	32	64	64	64	>64	>64	2	32	>64	2	>256	2	≤0.25	>256	>256	>512	≤2
EC-15-2-2	16	32	64	64	>64	>64	1	≤1	>64	2	>256	16	≤0.25	>256	>256	>512	>32
EC-15-2-5	64	64	64	64	8	>64	2	2	>64	>64	>256	64	≤0.125	>256	>256	>1024	>32
KP-15-2-6	32	32	>128	>128	32	64	4	≤1	1	8	>256	32	2	>256	>256	>512	> 32
EC-15-2-14	64	64	64	64	8	>64	2	≤1	>64	8	>256	32	0.25	>256	>256	>1024	> 32
SM-15-2-16	4	32	32	64	64	0.5	>16	8	2	4	>256	4	2	>256	>256	512	0.5
EC-15-2-24	32	64	64	64	>64	>64	1	≤1	>64	16	>256	16	≤0.125	>256	>256	>1024	> 32
EC-15-2-26	64	128	>64	>64	8	>64	2	512	>64	>64	>256	1	≤0.125	>256	>256	>1024	0.06
CF-15-2-29	32	32	>128	>128	>256	>64	2	≤1	256	32	>256	32	1	>256	>256	>512	>32
EC-15-2-35	32	32	>64	>64	>64	>64	2	≤1	>64	16	>256	16	≤0.125	>256	>256	>1024	> 32
EC-15-2-47	32	64	64	64	>64	>64	2	≤1	>64	16	>256	8	≤0.125	>256	>256	>1024	> 32
Pr-15-2-50	16	16	>1024	>64	8	16	-	256	-	>64	-	-	-	>256	-	>128	>32
EC-15-2-51	32	32	64	64	>64	>64	1	≤1	>64	64	>256	32	≤0.125	>256	>256	>1024	0.032
KP-15-2-52	64	64	≤0.25	>64	4	≤0.12	4	8	4	4	>256	4	0.25	>256	>256	>1024	0.125
EC-15-2-56	64	128	>64	>64	8	>64	2	2	8	8	>256	2	≤0.125	>256	>256	>1024	0.094
KP-15-2-62	64	64	1	>64	32	2	2	256	4	4	>256	4	0.25	>256	>256	>1024	> 32
EC-15-2-65	128	256	64	64	32	32	1	1024	>64	>64	>256	4	≤0.125	>256	>256	>1024	> 32
CF-15-2-98	32	32	32	128	16	8	4	1024	32	16	>256	32	1	>256	>256	>512	>32
KP-15-2-113	4	16	128	>128	4	16	>16	512	128	16	>256	4	2	>256	>256	512	> 32
EC-15-2-132	32	8	>128	>128	32	8	≤0.5	512	>64	64	4	16	0.5	>256	>256	16	> 32

EC-15-2-152	64	64	64	64	>64	>64	1	≤1	>64	4	>256	64		>256	>256	>512	0.38
EC-15-2-153	16	64	64	>128	256	2	2	16	512	8	>256	16	8	>256	>256	>512	>32
EC-15-2-159	64	512	64	64	16	>64	2	>1024	>64	>64	>256	64		>256	>256	>512	>32
CF-15-2-165	2	8	≤0.25	8	32	32	>64	>1024	8	8	>256	2	0.5	>256	>256	>512	≤2
ECL-16-5	16	16	64	>64	>128	1	2	16	>128	16	>256	128	2	>256	>256	>512	≤2
ECL-16-7	32	32	>64	>64	>128	>16	>256	64	>128	>128	>256	128	8	>256	>256	>512	>32
EC-16-10	16	16	0.5	>64	16	>16	≤0.5	4	2	2	>256	128	1	>256	>256	>512	>32
CF-16-17	8	16	64	>64	>128	>16	1	⩽1	2	4	>256	64	2	>256	>256	>512	>32
EC-16-20	32	32	>64	>64	128	>16	≤0.5	4	>128	>128	>256	128	0.5	>256	>256	>512	>32
KO-16-21	16	32	64	>64	128	>16	1	32	128	4	>256	4	2	>256	>256	>512	>32
EC-16-35	32	32	>64	>64	>128	>16	1	>1024	>128	>128	>256	64	2	>256	>256	>512	>32
EC-16-37	16	16	64	>64	16	16	1	4	>128	>128	>256	8	0.5	>256	>256	>512	>32
EC-16-52	32	32	>64	>64	>128	>16	1	1024	128	4	>256	64	2	>256	>256	>512	>32
KP-16-57	32	64	64	>64	4	>16	2	16	128	2	>256	32	1	>256	>256	>512	>32
CF-16-58	32	64	>64	>64	32	>16	2	≤1	>128	2	>256	>128	2	>256	>256	>512	≤2
EC-16-59	64	64	>64	>64	16	>16	1	≤1	128	4	>256	>128	0.5	>256	>256	>512	≤2
EC-16-60	32	16	>64	>64	4	>16	2	>1024	2	4	>256	2	0.5	>256	>256	>512	>32
CF-16-61	8	16	>64	>64	>128	16	2	1024	128	2	>256	8	2	>256	>256	>512	>32
EC-16-74	32	32	>64	64	16	>16	≤0.5	4	128	2	>256	128	2	>256	>256	>512	≤2
ECL-16-76	32	64	>64	>64	>128	>16	2	32	>128	>128	>256	>128	4	>256	>256	>512	≤2
EC-16-79	64	128	32	>64	8	16	≤0.5	8	128	4	>256	32	1	>256	>256	>512	>32

Abbreviations: MEM, meropenem; IPM, imipenem; ATM, aztreonam; CAZ, ceftazidime; CHL, chloramphenicol; CIP, ciprofloxacin; CST, colistin; FOS, fosfomycin sodium; GEN, gentamicin; AMK, amikacin; AMP, ampicillin; TET, tetracycline; TGC, tigecycline; CFX, cefoxitin; CFZ, cefazolin; TZP, Piperacillin–tazobactam; SXT, sulfamethoxazole/trimethoprim.

Supplementary Table 3. Basic information of 42 ST167 *bla*$_{NDM}$-bearing *E. coli* in NCBI nr database and this study.

Strain	BioSample	Host	Source	NDM-type	Region
51008369SK1	SAMN09381942	Dog	wound liquid	NDM-5	Switzerland
ECOL-18-VL-LA-PA-Ryan-0026	SAMN11230749	Dog	trach wash	NDM-5	USA
FDAARGOS_434	SAMN07312478	Homo sapiens	Rectal	NDM-5	Canada
CRE1493	SAMN06198937	Homo sapiens	Rectal Swab	NDM-5	Shenzhen
ECONIH6	SAMN05341196	Homo sapiens	swab	NDM-5	USA
AR_0150	SAMN04014991	-	-	NDM-5	-
AR_0151	SAMN04014992	-	-	NDM-5	-
AR_0149	SAMN04014990	-	-	NDM-7	-
AR_0162	SAMN04015003	-	-	NDM-7	-
Y5	SAMN04299569	Homo sapiens	urine	NDM-1	Zhejiang
AR435	SAMN07291528	-	-	NDM-1	-
M217	SAMD00059754	Homo sapiens	blood	NDM-5	Myanmar
CREC-629	SAMN07944243	Homo sapiens	urine	NDM-7	South Korea
ECWJ1	SAMN09435562	Homo sapiens	urine	NDM-5	Zhejiang
163	SAMN13951917	environment	-	NDM-5	Switzerland
164	SAMN13951916	environment	-	NDM-5	Switzerland
100	SAMN11872785	Homo sapiens	Urine	NDM-5	Italy
91	SAMN11872784	Homo sapiens	Stool	NDM-5	Italy
92	SAMN11872786	Homo sapiens	Stool	NDM-5	Italy
311	SAMN11872787	Homo sapiens	Stool	NDM-5	Italy
L665	SAMN10219249	Homo sapiens	feces	NDM-5	Zhejiang

L612	SAMN10219248	Homo sapiens	feces	NDM-5	Zhejiang
L610	SAMN10219247	Homo sapiens	feces	NDM-5	Zhejiang
L743	SAMN10219253	Homo sapiens	feces	NDM-5	Zhejiang
947385799	SAMN04011432	Homo sapiens	urine	NDM-5	South Africa
52148	SAMN14421542	Homo sapiens	urine	NDM-5	Czech Republic
EC-13-30	SAMN20568146	Homo sapiens	secreta	NDM-5	Zhengzhou
EC-13-31	SAMN20568144	Homo sapiens	blood	NDM-5	Zhengzhou
EC-13-49	SAMN20568151	Homo sapiens	urine	NDM-1	Zhengzhou
EC-14-2-9	SAMN20568171	Homo sapiens	sputum	NDM-5	Zhengzhou
EC-14-2-92	SAMN20568169	Homo sapiens	blood	NDM-5	Zhengzhou
EC-14-2-94	SAMN20568170	Homo sapiens	urine	NDM-5	Zhengzhou
EC-14-54	SAMN20568164	Homo sapiens	sanies	NDM-5	Zhengzhou
EC-15-2-1	SAMN20568196	Homo sapiens	urine	NDM-5	Zhengzhou
EC-15-2-159	SAMN20568201	Homo sapiens	urine	NDM-5	Zhengzhou
EC-15-2-26	SAMN20568194	Homo sapiens	urine	NDM-5	Zhengzhou
EC-15-2-5	SAMN20568172	Homo sapiens	sputum	NDM-5	Zhengzhou
EC-15-2-56	SAMN20568184	Homo sapiens	urine	NDM-5	Zhengzhou
EC-16-35	SAMN20568209	Homo sapiens	ascites	NDM-5	Zhengzhou
EC-16-59	SAMN20568214	Homo sapiens	tissue	NDM-5	Zhengzhou
EC-16-60	SAMN20568215	Homo sapiens	sputum	NDM-5	Zhengzhou
EC-16-7	SAMN20568203	Homo sapiens	urine	NDM-1	Zhengzhou

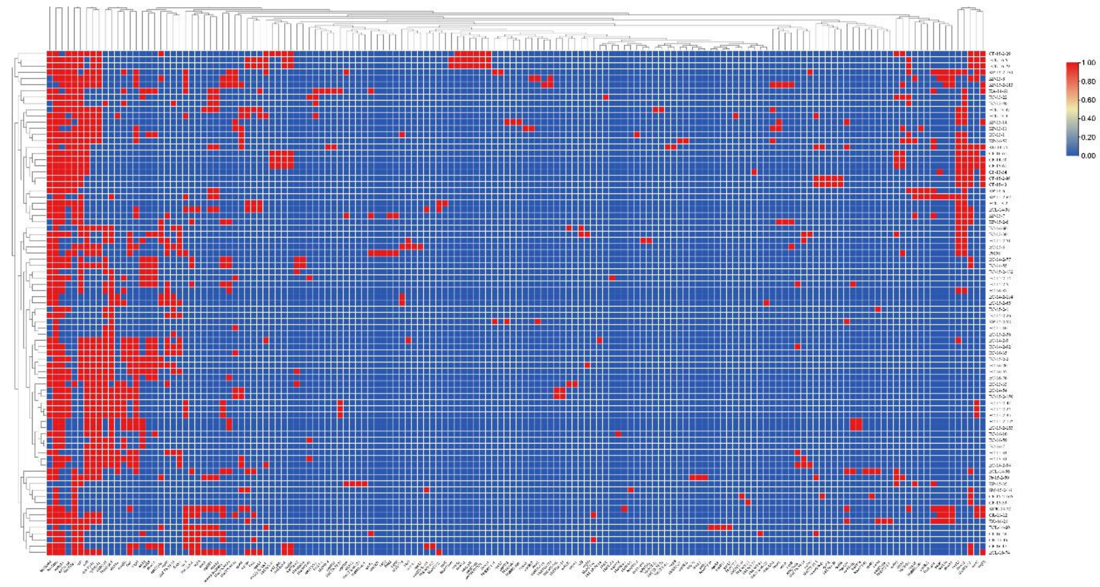

Supplementary Figure 1. Heat map of the resistance genes carried by *bla*$_{NDM}$-positive strains with whole genome sequences. Red represents the resistance gene carried in the strain.

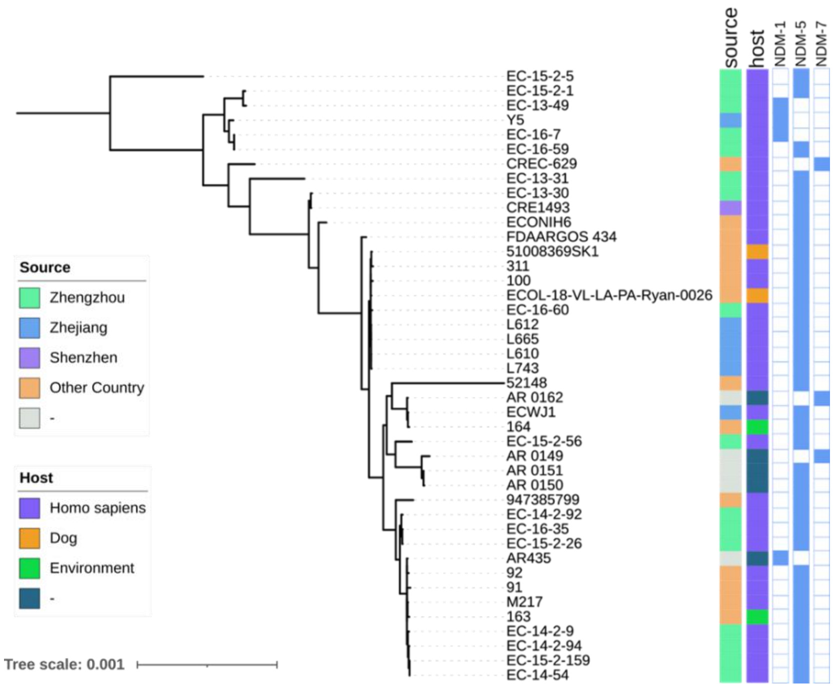

Supplementary Figure 2. Phylogenetic tree of the 42 ST167 *bla*$_{\mathbf{NDM}}$-positive *E. coli* strains analysed in this study and NCBI nr database. Branch lengths and scale bar represent number of nucleotide substitutions per site.

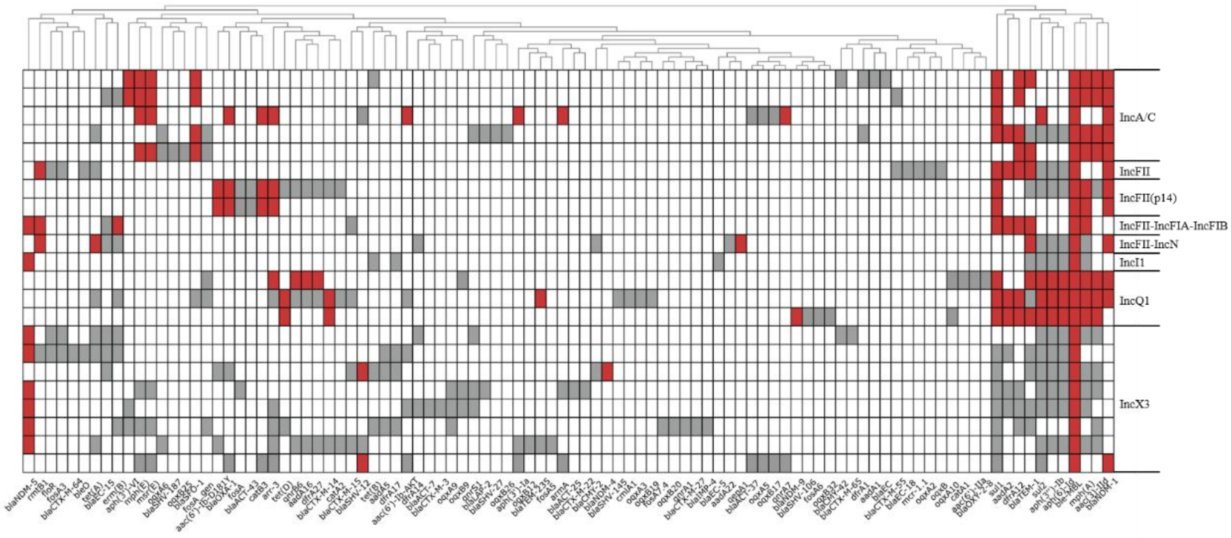

Supplementary Figure 3. Heatmap showing the distribution of AMR genes carried by 22 strains that were sequenced by Nanopore sequencing. Each row in the heat map represents one isolate and grouped by incompatibility types of the *bla*NDM-harbouring plasmids. Red squares indicate AMR genes located on the *bla*NDM-harbouring plasmids. The total red and gray squares in each row represent the AMR in the entire genome of each strain.

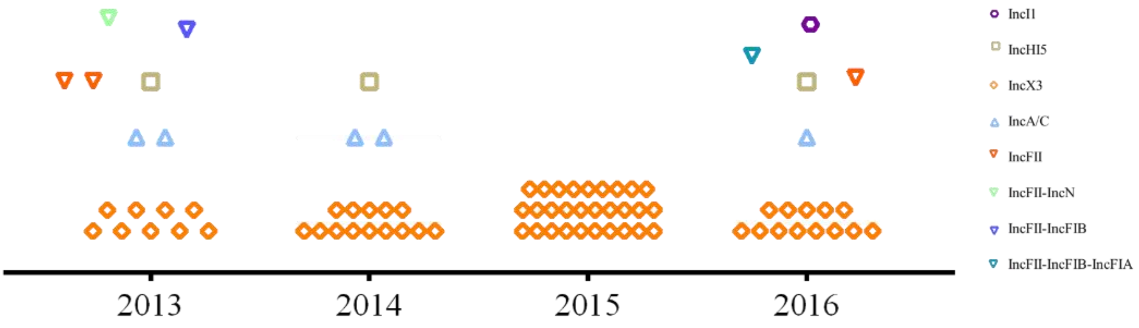

Supplementary Figure 4. Distribution of the replicon sequence types of *bla*$_{NDM}$-positive plasmids, based on the sampling dates. One point represents one sample.

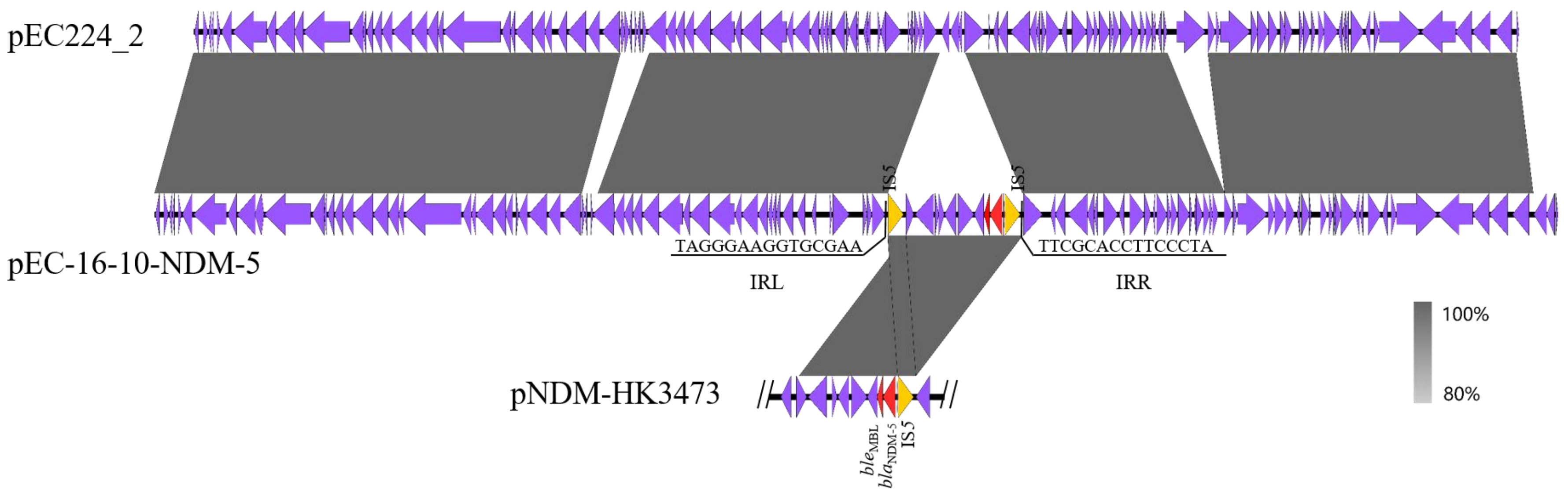

Supplementary Figure 5. Linear comparison of *bla*$_{NDM}$-bearing plasmids pEC-16-10-NDM5, pEC224_2 and pNDM-HK3473. The gray regions indicate the homologous region between plasmid regions.

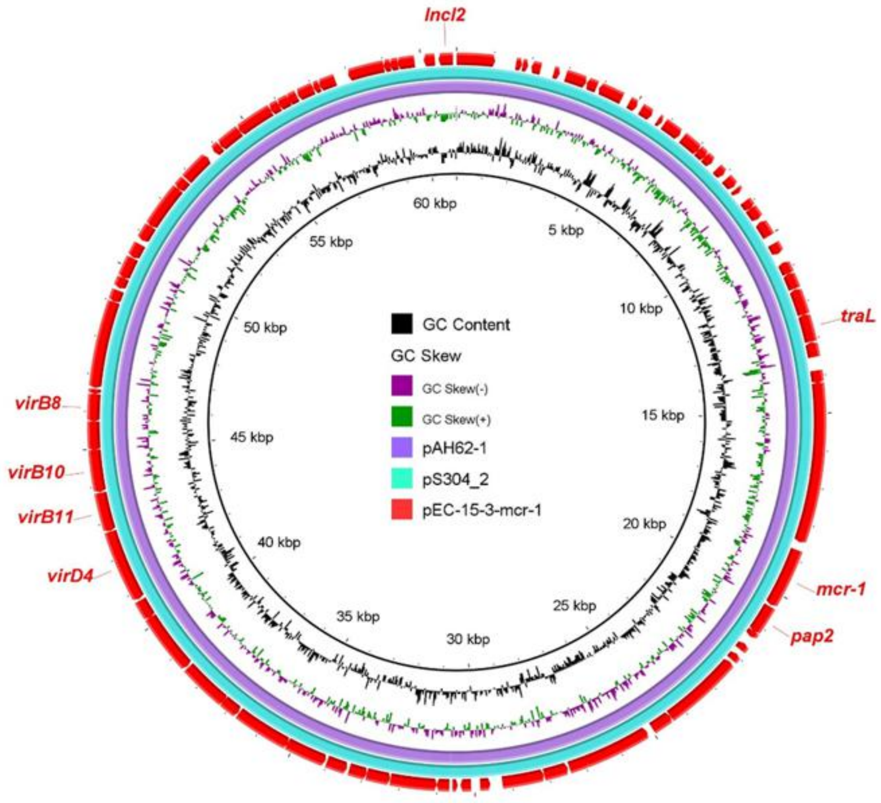

Supplementary Figure 6. Circular comparison between *mcr-1*-bearing IncI2 plasmids found in this study and in the online database. The *mcr-1*-bearing IncI2 plasmid pEC-15-3-mcr-1 was used as the reference in the outermost ring.

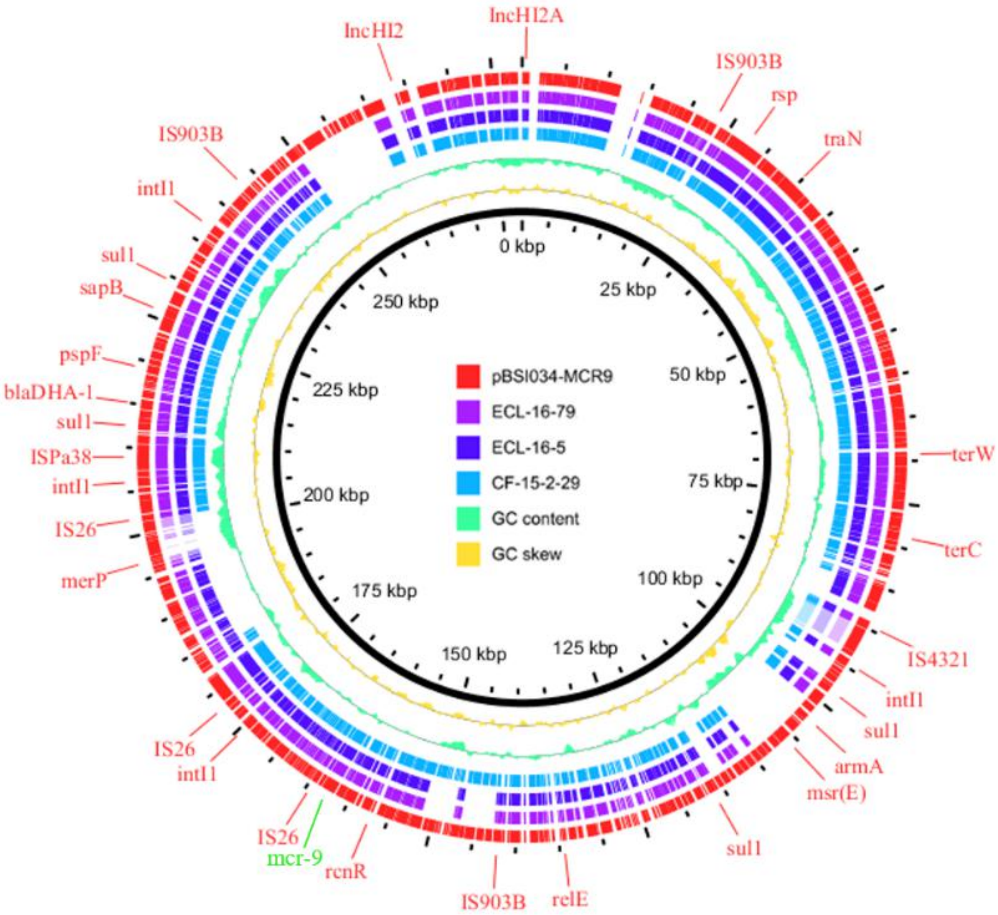

Supplementary Figure 7. Comparative analysis of the structures of the *mcr-9*-bearing region found in this study and pBSI034-MCR9 (NZ_MN937241).

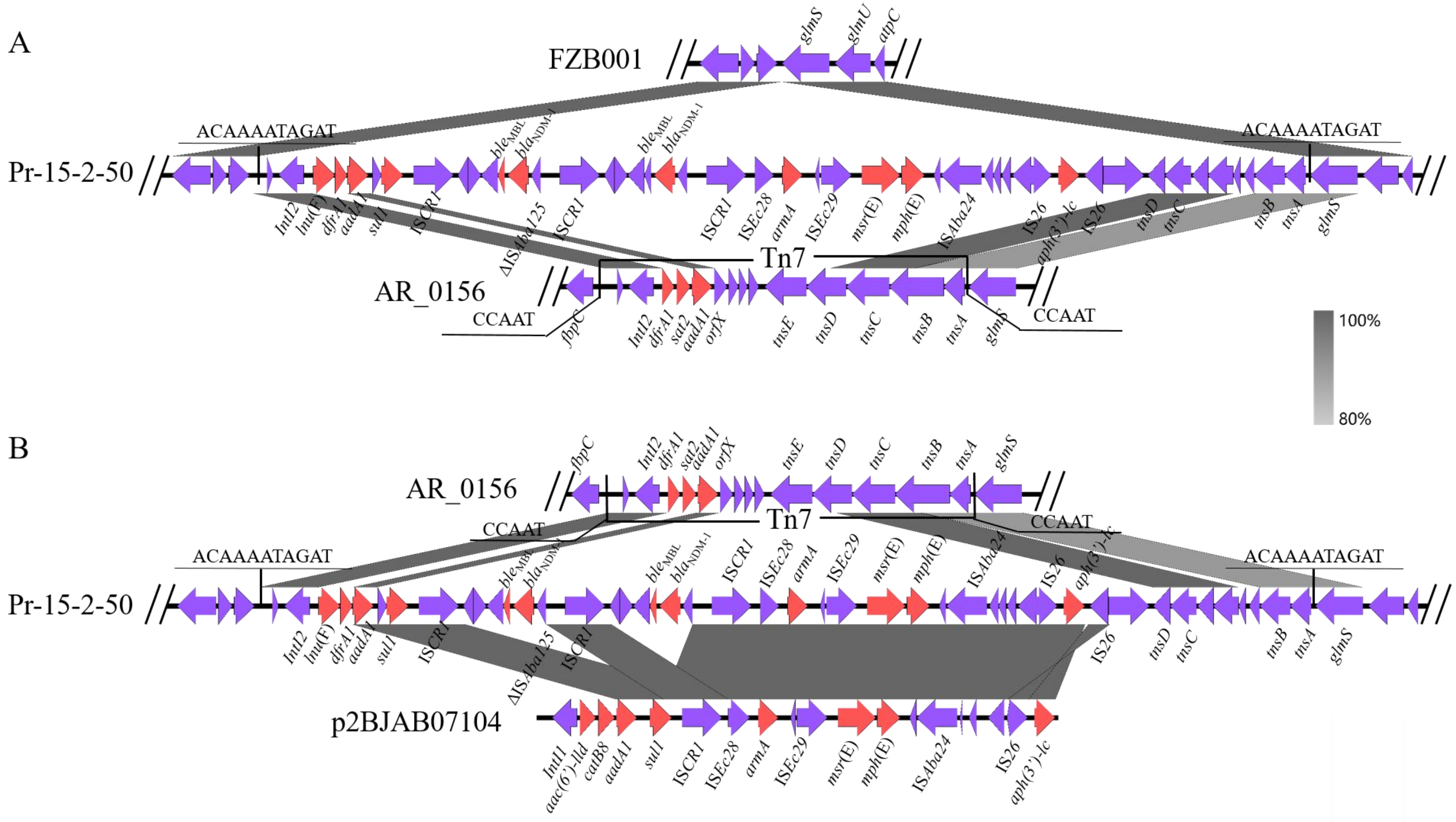

Supplementary Figure 8. Genetic structure of Pr-15-2-50. (A) Linear comparison between Pr-15-2-50, FZB001 and AR_0156. (B) Linear comparison between Pr-15-2-50, AR_0156 and p2BJAB07104. The gray regions indicate the homologous region between plasmid regions.
